# Improved albumin binding properties of Isoguvacine upon esterification as characterized by biophysical and computational tools

**DOI:** 10.1038/s41598-025-25957-7

**Published:** 2025-11-25

**Authors:** Yan Hong Ng, Muhamad Imam Muhajir, Rani Maharani, Unang Supratman, Jalifah Latip, Murni Nazira Sarian, Su Datt Lam, Shevin Rizal Feroz

**Affiliations:** 1 Department of Biological Sciences and Biotechnology, Faculty of Science and Technology, Universiti Kebangsaan Malaysia, Bangi, 43600 Selangor Malaysia; 2 Department of Chemistry, Faculty of Mathematics and Natural Sciences, Universitas Padjadjaran, Jatinangor, 45363 West Java Indonesia; 3 Central Laboratory, Universitas Padjadjaran, Jatinangor, 45363 West Java Indonesia; 4 Centre of Natural Products and Synthesis Studies, Faculty of Mathematics and Natural Sciences, Universitas Padjadjaran, Jatinangor, 45363 West Java Indonesia; 5 Department of Chemical Sciences, Faculty of Science and Technology, Universiti Kebangsaan Malaysia, Bangi, 43600 Selangor Malaysia; 6 Smart Material and Sustainable Product Innovation (SMatSPIn) Research, Universiti Kebangsaan Malaysia, Bangi, 43600 Selangor Malaysia; 7 Institute of Systems Biology (INBIOSIS), Universiti Kebangsaan Malaysia, Bangi, 43600 Selangor Malaysia; 8 Department of Applied Physics, Faculty of Science and Technology, Universiti Kebangsaan Malaysia, Bangi, 43600 Selangor Malaysia; 9 Structural Biology and Protein Engineering Research Group, Universiti Kebangsaan Malaysia, Bangi, 43600 Selangor Malaysia; 10 Center for Global Health Research (CGHR), Saveetha Institute of Medical and Technical Sciences (SIMATS), Saveetha Medical College, Saveetha University, Chennai, 602105 India

**Keywords:** Epilepsy, Isoguvacine, Esterification, Human serum albumin, Protein–ligand interaction, Biochemistry, Biophysics, Chemistry, Computational biology and bioinformatics, Drug discovery

## Abstract

The prevalence of epilepsy, affecting millions across all age groups, has intensified the need for novel therapeutic interventions. Despite advances in antiseizure medications (ASMs), challenges persist, particularly drug resistance and significant psychiatric and behavioral adverse effects. Isoguvacine (IGV), a potent γ-aminobutyric acid type A receptor agonist, holds promise for epilepsy treatment but suffers from poor blood–brain barrier (BBB) permeability due to its zwitterionic nature. To address this limitation, two ester prodrugs: **E7** (heptyl 1,2,3,6-tetrahydropyridine-4-carboxylate) and **E14** (tetradecyl 1,2,3,6-tetrahydropyridine-4-carboxylate) were synthesized to enhance BBB penetration and central nervous system (CNS) delivery. This study investigates the interactions of IGV and its derivatives with human serum albumin (HSA), a key plasma transport protein, using a suite of biophysical and computational techniques. UV spectroscopic analysis confirmed protein–ligand complexation, with **E7** and **E14** exhibiting enhanced binding compared to IGV. Isothermal titration calorimetry revealed that **E14** demonstrated superior binding affinity (*K*_*a*_ = 2.42 × 10^6^ M⁻¹) compared to **E7** (*K*_*a*_ = 3.41 × 10^4^ M⁻¹), while IGV showed negligible interaction. Atomic force microscopy and circular dichroism spectroscopy indicated that the enhanced hydrophobicity of **E7** and **E14** promoted stable protein–ligand interactions without perturbing the secondary and tertiary structures of HSA. Molecular docking studies corroborated the superior binding affinity of **E14** towards HSA, while molecular dynamics simulations confirmed the enhanced stability of the **E14**–HSA complex. These results highlight the potential of **E14** as a promising prodrug candidate for CNS-targeted epilepsy therapy and underscore the importance of albumin binding in modulating drug bioavailability and distribution.

**Supplementary Information:**

The online version contains supplementary material available at 10.1038/s41598-025-25957-7.

## Introduction

Epilepsy, a complex neurological disorder characterized by recurrent seizures due to abnormal cerebral electrical activity, has been documented since ancient times and continues to pose significant challenges in modern medicine^1^. The global burden of this condition is substantial, affecting approximately 50 million individuals worldwide, with an annual incidence of 5 million new cases. Notable epidemiological data indicates a disproportionate impact on rural populations in low- and middle-income countries, where nearly 80% of cases are reported^2^. Current therapeutic options encompass various antiseizure medications (ASMs), with ganaxolone being the most recent addition to the FDA-approved treatments in March 2022^3^. These medications primarily function by suppressing neurotransmission to maintain the excitation-inhibition balance in the brain. Nevertheless, the lack of new therapeutic innovations, coupled with alarming rates of drug resistance and adverse effects among patients, highlights the pressing need for novel treatment strategies.

Central to the pathophysiology of epilepsy and its treatment are γ-aminobutyric acid type A receptors (GABA_A_Rs), which play a crucial role in mediating inhibitory neurotransmission. The complexity of GABA_A_R, featuring multiple allosteric and orthosteric binding sites, presents promising opportunities for therapeutic intervention^4^. Notably, approximately one-third of FDA-approved ASMs target GABA_A_R-mediated mechanisms, either through direct receptor modulation or by affecting GABA availability, underlining the continued significance of GABA_A_R in epilepsy management^5^.

Isoguvacine (IGV), a highly potent and selective agonist of GABA_A_Rs, has gained attention as a promising candidate for developing new epilepsy treatments^6^. Beyond its application as a common antiseizure agent in experimental animal models, IGV has also demonstrated efficacy in addressing other neurological conditions such as autism^7^. However, despite its pharmacological potential, IGV faces a critical drawback: its zwitterionic nature restricts blood-brain barrier (BBB) permeability. While IGV can still modulate seizure activity by targeting peripheral GABA_A_Rs or regions with less restrictive BBB (e.g.: circumventricular organs), its poor central nervous system (CNS) penetration limits efficient seizure control^5^.

**Fig. 1:**
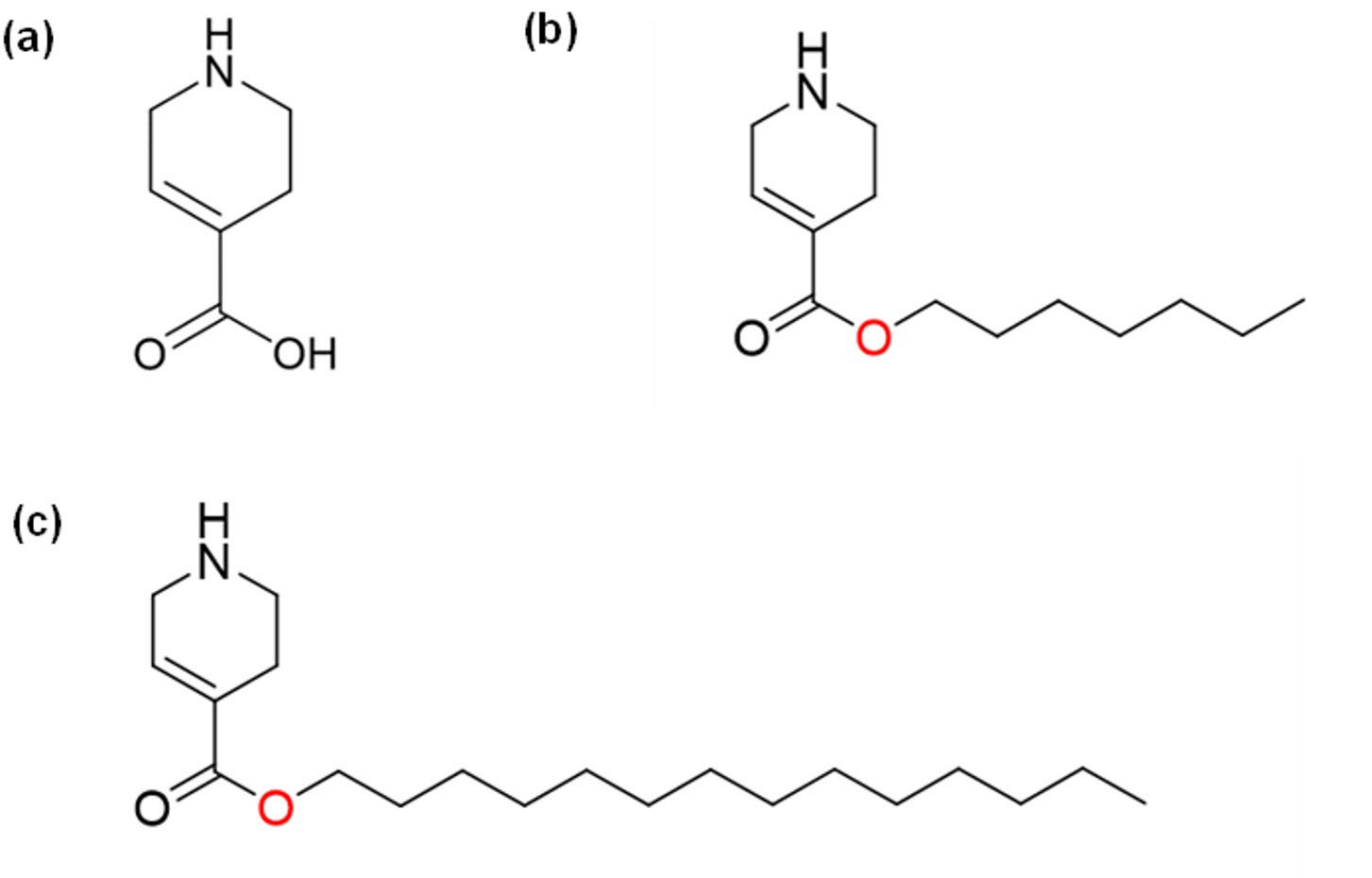
Chemical structures of (**a**) IGV, (**b**) **E7**, and (c) **E14**.

To overcome this challenge, we have designed and synthesized two ester prodrug derivatives of IGV, i.e.: **E7** (heptyl 1,2,3,6-tetrahydropyridine-4-carboxylate) and **E14** (tetradecyl 1,2,3,6-tetrahydropyridine-4-carboxylate) (Fig. 1; synthesis and characterization details are depicted in Figs. S1–S3). These derivatives are predicted to effectively cross the BBB (Fig. S4), where they are expected to be metabolized into the active IGV, enabling targeted antiseizure effects within the CNS while minimizing peripheral side effects. However, a key limitation in fully exploring their therapeutic value lies in the insufficient understanding of their interactions with human plasma transport proteins.

The therapeutic efficacy of drugs is ultimately determined by their pharmacodynamic and pharmacokinetic properties, which are influenced by their structural characteristics and interaction with plasma transport proteins^8,9^. The main transport protein in the circulatory system, human serum albumin (HSA), is known to transport a wide range of ligands and exhibits varying interactions with different drugs, revealing distinct affinity, thermodynamic parameters, intermolecular forces, and binding sites^10^.

In this study, a multi-modal analytical approach was employed to elucidate the molecular interactions of HSA with IGV and its ester derivatives. UV-Vis spectroscopic analysis was utilized to characterize the formation of protein-ligand complexes, while thermodynamic parameters and binding constants were quantitatively determined through isothermal titration calorimetry (ITC). Protein surface topology alterations were examined using atomic force microscopy (AFM), and changes in protein secondary and tertiary structures were evaluated via circular dichroism (CD) spectroscopy. In silico studies encompassed molecular docking simulations to identify potential binding sites of the ligands on HSA. Subsequently, molecular dynamics (MD) simulations were performed to assess the temporal evolution and stability of these protein–ligand complexes. This integrated experimental and computational approach provides mechanistic insights into the molecular basis of HSA–ligand interactions, crucial for evaluating the therapeutic potential of these novel IGV derivatives.

## Materials and methods

### Materials

HSA and IGV (≥ 98% purity for both) were purchased from Sigma-Aldrich Co. (St Louis, MO, USA). **E7** and **E14** were synthesized through a two-step Steglich esterification process, as previously reported^11^ (Fig. S1). The chemicals used in the synthesis process included 1-(tert-butoxycarbonyl)-1,2,3,6-tetrahydropyridine-4-carboxylic acid (Boc-IGV) (cat. no. A684843, purity ≥ 98%), obtained from Alfa Chemical Co. Ltd. (Zhengzhou, China); 1-ethyl-3-(3-dimethylaminopropyl)carbodiimide hydrochloride (EDC∙HCl) (cat. no. 03449, purity ≥ 99%), 4-dimethylaminopyridine (DMAP) (cat. no. 107700, purity ≥ 99%), and trifluoroacetic acid (TFA) (cat. no. 808260, purity ≥ 99%) purchased from Sigma-Aldrich (St. Louis, MO, USA); and the alcohol substrates 1-heptanol (cat. no. 55120, purity ≥ 99%) and 1-tetradecanol (cat. no. 8081460100, purity ≥ 97%) supplied by Merck KGaA (Darmstadt, Germany). All other chemicals were of analytical purity.

### Preparation of protein and ligand solutions

All solutions were prepared using 10 mM sodium phosphate buffer, adjusted to pH 7.4 using 0.1 M NaOH. HSA crystals were dissolved in the buffer to make a stock solution whose concentration was verified spectrophotometrically using an extinction coefficient of 35,219 M^− 1^ cm^− 1^ at 280 nm^12^, before stored at 4 °C. Similarly, the IGV stock solution was prepared in the same buffer; while its derivatives, **E7** and **E14**, were initially dissolved in methanol before dilution with the same buffer to prepare their respective stock solutions. Subsequently, all working solutions were obtained through serial dilution using the above buffer.

### UV spectroscopy

To investigate the occurrence of complexation between HSA and the ligands, absorption measurements were recorded using a Shimadzu UV-1800 spectrophotometer with a pair of quartz cuvettes (1 cm path length) in the 200–350 nm region. Sample spectra were obtained by subtracting the spectra of the individual ligands from the corresponding HSA–ligand mixtures. Measurements were performed at a fixed HSA concentration (15 µM) with varying ligand concentrations of 7.5 µM, 15 µM, and 30 µM.

### Isothermal titration calorimetry

The binding between HSA and three compounds (IGV, **E7**, and **E14**) was investigated using isothermal titration calorimetry (ITC) with a Nano ITC microcalorimeter (TA Instruments, New Castle, DE, USA) at 25 °C. The ligands were initially dissolved in methanol to prepare 3 mg/mL stock solutions, while HSA was prepared at 2 mg/mL in 10 mM sodium phosphate buffer (pH 7.4). Working solutions were then prepared by diluting the stocks with the same buffer to achieve a final methanol concentration of approximately 3%. To eliminate air bubbles, all solutions were degassed for 10 min under vacuum. The ITC experiments were performed by loading the sample cell with HSA solution (20 µM) and the reference cell with deionized water. The ligand solution (200 µM) was loaded into a 50 µL injection syringe. The titration protocol consisted of 16 successive injections of ligand solution (2.5 µL each) at 200 s intervals, with continuous stirring at 200 rpm. To account for the background heat effects from mixing and dilution, control experiments were conducted by titrating the ligand solutions into buffer under identical conditions. The thermodynamic parameters were determined using NanoAnalyze software (version 3.3.0).

### Atomic force microscopy

Surface topographical analysis of HSA in the absence and presence of the ligands was conducted using a Park Systems NX-10 atomic force microscope (Suwon, Korea). The instrument specifications included a 15 μm scan range, 0.015 nm resolution, 0.03 nm position detector noise (at 1 kHz bandwidth), and a resonant frequency of 10.5 kHz. Sample preparation began with mounting freshly cleaved mica sheets (1 cm × 1 cm) onto glass slides. Two sample sets were prepared: free HSA solution (1.5 µM) and 1.5 µM HSA–ligand complexes at a 1:1 molar ratio. For each analysis, 30 µL of sample was carefully deposited onto the mica substrate and allowed to adsorb for 15 min. Unbound molecules were gently washed away using 10 mM sodium phosphate buffer, and samples were left overnight. Topographical imaging was performed over a 4 μm × 4 μm area, with both 2D and 3D visualizations generated using XEI software (version 4.3.4).

### Circular dichroism spectroscopy

A Jasco J-815 spectropolarimeter (Tokyo, Japan), equipped with a Peltier temperature controller, was utilized to acquire far-UV and near-UV circular dichroism (CD) spectra of HSA at 25 °C under continuous nitrogen gas flow. Far-UV CD signals in the range 200–250 nm was recorded using a 1 mm path length cuvette, while near-UV CD spectra in the 250–300 nm region were measured with a 10 mm path length cuvette. The scan speed was set to 100 nm/min, with a data pitch of 1.0 nm and a response time of 1 s. Each sample was scanned three consecutive times to generate a single CD spectrum, and the resulting data was smoothed using the Savitzky-Golay method. The HSA concentration was maintained at 1 µM and 10 µM for far-UV and near-UV measurements, respectively. Protein spectra were recorded in the presence of IGV and its derivatives at protein to ligand molar ratios of 1:0, 1:1, 1:2, and 1:3. The BeStSel server was then used to determine the secondary structure composition of HSA based on the obtained spectra. The server is capable of predicting the abundance of various secondary structure elements, including α-helix, β-antiparallel, β-parallel, and β-turns in a given protein^13^.

### Statistical data analysis

All quantitative data are presented as mean ± standard deviation from three independent replicates. Statistical analyses were carried out using GraphPad Prism, version 9.0 (GraphPad Software, San Diego, CA, USA).

### Molecular docking

The pK_a_ values of the ligands were first determined using the ChemAxon Chemicalize platform, which employs a knowledge-based algorithm incorporating chemical fragment data and physicochemical rules to estimate ionization constants^14^. These predicted pK_a_ values were then used to assign the appropriate protonation states of the ligands under physiological conditions prior to molecular docking analysis.

Protein and ligand structures were prepared using AutoDockTools version 1.5.7^15^. Protein conformers were obtained from the Protein Data Bank (PDB) (www.rscb.org) while the 2D structures of the ligands were created in ChemDraw Professional 16.0, and their corresponding 3D structures were generated using Chem3D Professional 16.0 (PerkinElmer Informatics, 2016). These structures were then refined through energy minimization with the MM2 force field^16^. For receptor preparation, the crystal structure of the protein was processed by removing ligands and water molecules, adding polar hydrogens, and assigning Kollman unified partial charges. Ligand preparation involved defining rotatable bonds, merging nonpolar hydrogens, and applying Gasteiger charges. The protein and ligand structures were converted to .pdbqt format for docking analysis.

To validate the reliability of the docking protocol, myristic acid (MYR), a well-characterized endogenous ligand that binds at the FA5 fatty acid binding site located within subdomain IIIB of HSA, was initially docked to this site. Subsequently, IGV and its derivatives were docked into the same binding pocket to ensure consistent and comparative analysis. For specific-site docking, grid maps with dimensions of 50 × 50 × 50 Å and a grid spacing of 0.375 Å were generated. An X-ray crystallographic structure of the protein (PDB ID: 1BM0) at a resolution of 2.5 Å was used, and the grid coordinates were set to x = 17.370, y = 19.306, and z = − 1.367. For blind docking, grid maps with dimensions of 126 × 126 × 126 Å and a grid spacing of 0.508 Å were generated, centered at coordinates x = 29.610, y = 31.792, and z = 23.486.

Docking simulations of HSA with ligands were performed using AutoDock4.2^17^, employing the Lamarckian genetic algorithm for the search process. Parameters included 100 search runs, a population size of 150, a maximum of 27,000 generations, a mutation rate of 0.02, a crossover rate of 0.8, and elitism of 1. Clustering analysis was performed with an RMSD tolerance of 2.0 Å, and the docking pose with the lowest binding energy was selected for further study. The best binding conformation was then analyzed using AutoDockTools and visualized using UCSF Chimera version 1.19^18^, while molecular interactions were determined using BIOVIA Discovery Studio Visualizer version 4.5 (BIOVIA, Dassault Systèmes, 2021).

### Molecular dynamics simulation

Molecular dynamics (MD) simulations of the HSA–ligand interactions were conducted utilizing GROMACS version 2022.3 software package^19^. The study implemented the GROMOS54a7 force field in conjunction with the single-point charge (SPC) water model for simulation parameters^20^. Topology and parameter files of the ligands were established using ATB 3.0 (Automated Topology Builder Version 3.0)^21^. Each molecular complex was positioned within a cubic simulation box, maintaining a 1 Å buffer zone to ensure adequate space for dynamics. System electroneutrality was achieved through the introduction of sodium and chloride counter ions, while maintaining appropriate ionic strength conditions. The initial energy minimization phase employed the steepest descent algorithm, executing 50,000 steps to resolve potential steric conflicts and achieve optimal energy configurations. The system then underwent sequential equilibration procedures: first, a 100 ps NVT equilibration to achieve temperature stability at 298 K, followed by a 100 ps NPT equilibration to establish pressure equilibrium at 1 atm. Throughout the equilibration process, strategic position restraints were implemented to maintain structural integrity. Additionally, the system was configured to couple the ligand and protein temperatures through the creation of a unified index file using GROMACS’ gmx make_ndx functionality.

Following equilibration, MD simulations were executed over a 100 ns timeframe, with structural coordinates recorded at 10 ps intervals across all systems. Each simulation was performed in triplicate to ensure reproducibility and provide statistically reliable results. The resulting trajectory data underwent comprehensive analysis using various GROMACS analytical tools. Structural stability was evaluated through RMSD calculations using gmx rms, while atomic fluctuations were assessed via RMSF analysis using gmx rmsf. The gmx gyrate tool provided insights into structural compactness through radius of gyration (Rg) measurements, and hydrogen bonding patterns were examined using gmx hbond. Visual analysis of the simulation results was performed using VMD, while graphical data representation was accomplished using the GRACE software package version 5.1.22.

## Results and discussion

### Formation of HSA–ligand complex

**Fig. 2:**
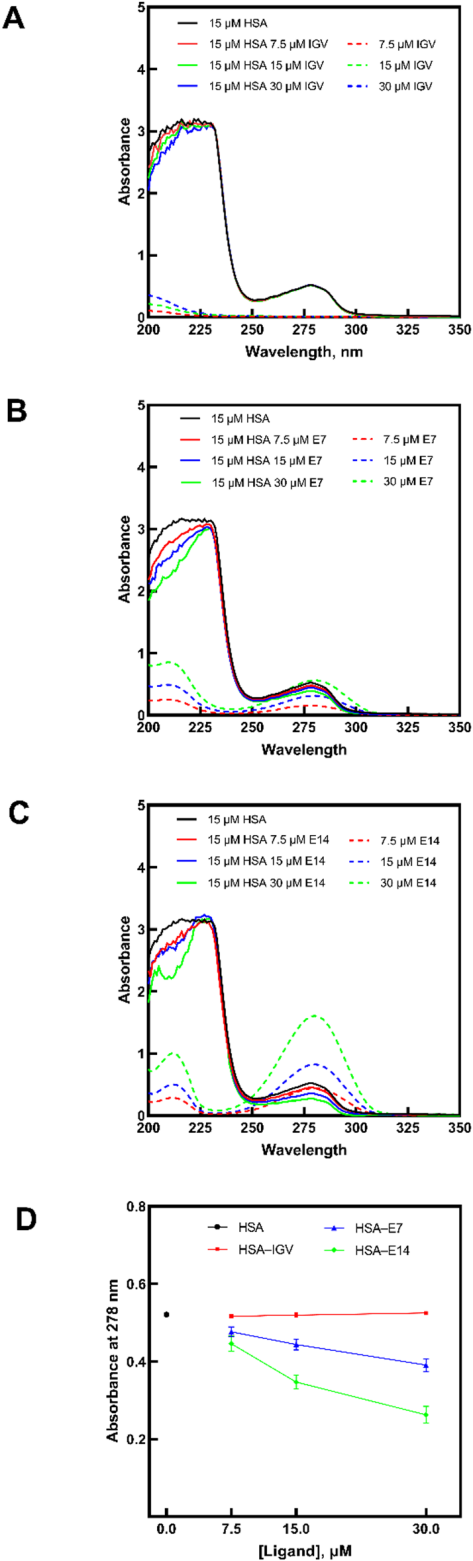
UV absorption spectra of HSA (15 µM) in the absence and presence of increasing concentrations (7.5, 15, and 30 µM) of (**A**) IGV, (**B**) **E7**, and (**C**) **E14**. Solid lines represent free HSA and HSA–ligand complexes (after subtracting the free ligand spectra), while dashed lines show the spectra of free ligands at the same concentrations. (**D**) Absorbance at 278 nm of HSA in the absence and presence of the ligands plotted as mean ± standard deviation.

As shown in Fig. 2, HSA exhibits two characteristic absorption peaks: one near 222 nm, attributed to peptide carbonyl n → π* transitions, and another at 278 nm, primarily arising from π → π* transitions of the aromatic amino acids (Trp, Tyr, and Phe) within their phenyl rings^22,23^. In comparison, IGV (Fig. 2A) shows only a gradual increase in absorbance between 200 and 210 nm, indicating localized electronic transitions confined to this lower-wavelength region. By contrast, **E7** (Fig. 2B) and **E14** (Fig. 2C) display two distinct absorption bands at 210 nm and 280 nm. The higher-energy band at 210 nm corresponds to π → π* transitions within the conjugated tetrahydropyridine ring system, consistent with nitrogen-containing heterocyclic chromophores, while the weaker band at 280 nm originates from n → π* transitions of the ester carbonyl group, characteristic of ester functionalities^24^. These dual peaks highlight electronic contributions from both the heterocyclic ring and carbonyl chromophores. Overall, these observations are consistent with previous reports on HSA interactions with other conjugated and heteroaromatic compounds^24–26^.

The absorption of the HSA–ligand complexes increased directly proportional to the ligand concentration. This hyperchromic shift could either indicate ligand-induced microenvironmental changes around the chromophores, or simply absorbance attributed to the presence of free ligands. To distinguish these effects, difference spectra were obtained by subtracting the spectra of the free ligands from those of the corresponding HSA–ligand complexes (Fig. 2A–C). The resulting changes in absorption at 278 nm are summarized in Fig. 2D, highlighting the influence of ligand concentration on the spectral features of HSA.

For IGV, subtraction of the free ligand absorbance from the HSA–ligand complex showed nearly overlapping spectra at 278 nm, indicating minimal interaction between HSA and IGV. On the other hand, the spectra of the HSA–**E7** and HSA–**E14** complexes, after subtraction of the free ligands, displayed a clear hypochromic trend rather than the initial hyperchromic effect, providing evidence of albumin–ligand binding. This observation suggests HSA–ligand complexation with a ground state absorbance that is lower than that of the unbound protein. This reduction in absorbance is associated with changes in the electronic configuration of the complex, likely due to energy transfer mechanisms^27,28^. The more pronounced HSA interaction observed with **E14** may be attributed to its longer hydrocarbon chain which facilitates binding to the fatty acid binding sites of the protein^29,30^.

### Binding and thermodynamic characteristics of HSA–ligand interactions

Analysis of HSA–ligand interactions through the direct measurement of heat change during binding events was performed using ITC. This allows for the precise determination of key thermodynamic parameters, such as the association constant (*K*_a_), changes in enthalpy (Δ*H*) and entropy (Δ*S*), as well as the binding stoichiometry (*n*)^31^. Integration of these parameters enables mechanistic interpretation of non-covalent interactions, including hydrogen bonding, van der Waals forces, and hydrophobic effects, together with conformational changes that dictate the binding process.

**Fig. 3:**
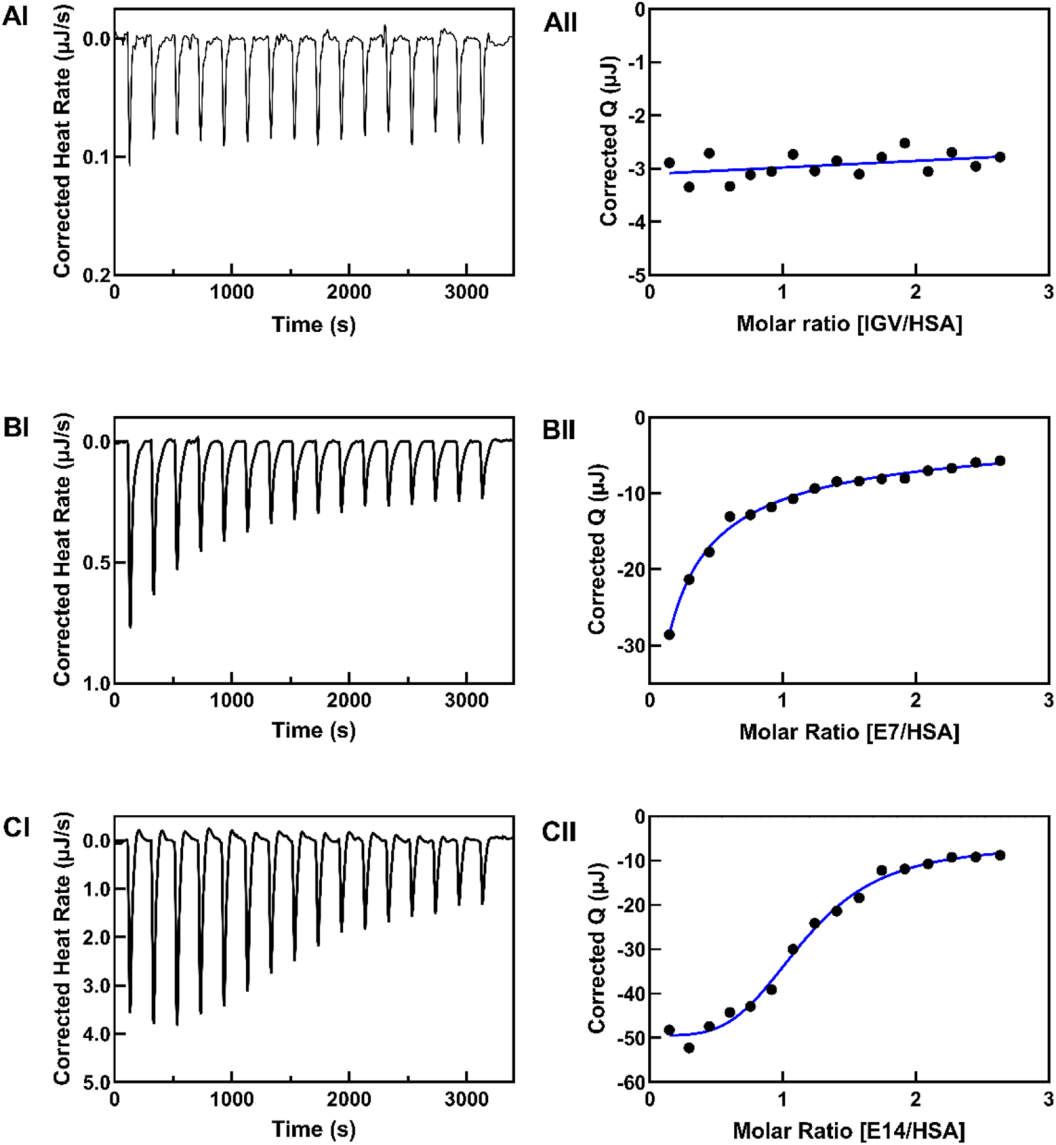
ITC profiles of the interaction between HSA and (**A**) IGV, (**B**) **E7**, and (**C**) **E14**. (I) Calorimetric response during successive injections of 200 µM ligand into 20 µM HSA. (II) Heat released during titration plotted against ligand/protein molar ratio.

The interactions of HSA with IGV and its derivatives were characterized by marked differences in binding affinity and thermodynamic characteristics. The parent compound IGV exhibited binding that was too weak to be quantified under the experimental conditions, indicating minimal interaction with HSA (Fig. 3A). In contrast, both **E7** and **E14** are bound strongly to HSA, exemplifying how structural modifications can substantially influence protein binding properties (Fig. 3B and C). As listed in Table 1, E14 demonstrated a high affinity towards HSA with a *K*_a_ of (2.42 ± 1.21) × 10^6^ M⁻¹, approximately two orders of magnitude higher than did **E7** (*K*_a_ = (3.41 ± 0.76) × 10^4^ M^− 1^). This substantial difference in binding affinity is reflected in the change in Gibbs free energy, where **E14** showed a more favorable Δ*G* (− 31.79 kJ mol^− 1^) compared to **E7** (− 25.83 kJ mol^− 1^). Such a range of *K*_a_ values is generally regarded as appropriate for efficient drug transport through the bloodstream and controlled release at target sites, as demonstrated in previous studies involving therapeutic compounds bound to HSA^32,33^. These observations further indicate that the structural characteristics of **E14** are particularly well-suited for interaction with the binding sites of HSA.

Both derivatives exhibited exothermic binding processes, with **E14** showing a more negative enthalpy change (Δ*H* = − 32.64 kJ mol^− 1^) compared to **E7** (Δ*H* = − 23.64 kJ mol^− 1^). The greater magnitude of negative enthalpy for **E14** indicates stronger intermolecular forces in its interaction with HSA, possibly involving hydrogen bonding networks and van der Waals interactions. However, the observation of positive entropy changes for both compounds (ΔS = 7.33 J mol^− 1^ K^− 1^ for **E7**, and 14.61 J mol^− 1^ K^− 1^ for **E14**) suggests that the binding process is predominantly driven by hydrophobic forces rather than hydrogen bonds and van der Waals forces, which typically show simultaneous negative enthalpy and entropy changes^34,35^. This increase in system disorder could be attributed to the release of water molecules from the binding interface and increased conformational flexibility in certain regions of the protein–ligand complex. Hence, the HSA–ligand interactions appear to be predominantly driven by hydrophobic interactions, supported by enthalpic contributions, indicating a complex interplay of specific molecular forces.

The binding stoichiometry values differed between the two derivatives, with **E7** exhibiting approximately two binding sites (*n* = 1.68) and **E14** showing higher binding capacity with approximately three binding sites (*n* = 2.50) on HSA. These findings suggest the availability of multiple binding sites on HSA for these compounds, which is sensible considering the fact HSA possesses several fatty acid binding sites that can accommodate the hydrocarbon tails of **E7** and **E14**.

**Table 1:** Binding and thermodynamic parameters for the interaction of **E7** and **E14** with HSA.

Parameter	**E7**	**E14**
Binding affinity, *K*_a_/ M^− 1^	(3.41 ± 0.76) × 10^4^	(2.42 ± 1.21) × 10^6^
Enthalpy change, Δ*H*/ kJ mol^− 1^	−23.64	−32.64
Entropy change, Δ*S*/ J mol^− 1^ K^− 1^	7.33	14.61
Stoichiometry, *n*	1.68	2.50
Gibbs free energy change, Δ*G*/ kJ mol^− 1^	−25.83	−31.79

### Topological changes in HSA induced by ligand binding

Atomic force microscopy detects subtle topological changes on protein surfaces resulting from ligand interactions^36^. Previous studies have demonstrated that ligand binding can significantly alter protein topology, impacting surface morphology and promoting aggregation^37–39^.

**Fig. 4:**
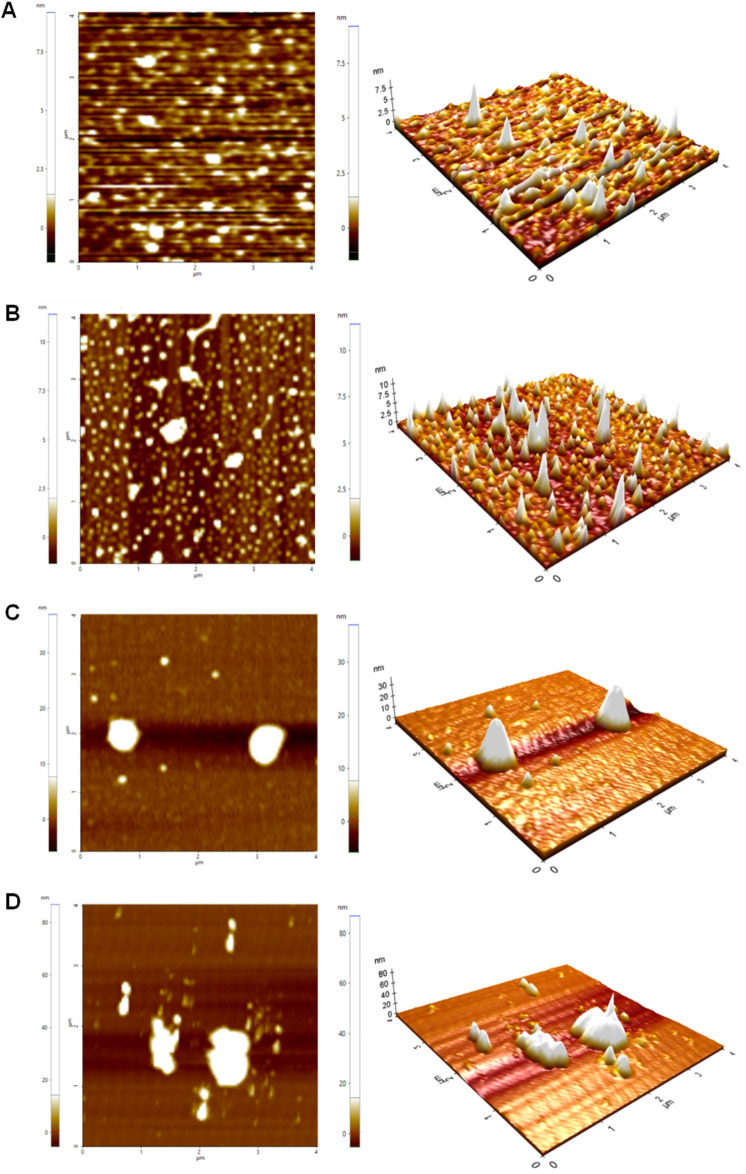
2D and 3D AFM topographic images of (**A**) unbound HSA, (**B**) HSA–IGV, (**C**) HSA–**E7**, and (**D**) HSA–**E14**. The samples were analyzed using tapping mode with a scan area of 4 μm × 4 μm.

AFM analysis revealed notable topological differences in the surface morphology of HSA upon interaction with **E7** and **E14**. The unbound HSA (Fig. 4A) displayed a smooth and consistent surface with minimal aggregation, characterized by a peak height of approximately 7.5 nm, reflecting the intact and native structure of the protein. This is consistent with previous AFM observations of native HSA, which demonstrate its uniform and compact morphology with minimal aggregation^25,40–42^. The presence of IGV (Fig. 4B) resulted in a surface morphology that closely resembled that of unbound HSA, with only a slight increase in surface height to 10 nm and minimal changes in surface texture. These observations suggest that IGV interacts weakly with HSA and does not alter the overall structural integrity or surface properties of the protein.

In contrast, noticeable topological changes were observed with the addition of **E7** and **E14**. The HSA–**E7** complex (Fig. 4C) exhibited peak heights of up to 30 nm, along with pronounced irregularities and larger protrusions on the surface. Likewise, the HSA–**E14** complex (Fig. 4D) showed similar features, with peak heights increasing up to 80 nm. This indicates stronger interactions between **E7/E14** and HSA, likely driven by hydrophobic binding, which promote aggregation and clustering of non-polar regions. Therefore, the observed similarity between free HSA and HSA–IGV, as opposed to the pronounced changes seen in the HSA–**E7** and HSA–**E14** complexes, demonstrates the influence of ligand hydrophobicity on protein–ligand interactions.

### Effect of ligand interactions on the secondary and tertiary structures of HSA

CD spectroscopy has been widely used to study protein structural changes in response to interactions with ligands^43,44^. Far-UV CD spectroscopy typically involves measuring the CD signal in the wavelength range of 200–250 nm. This corresponds to the absorbance of peptide bonds due to the n → π* transition of amides and is sensitive to the presence of secondary structures such as alpha helices, beta sheets, and random coils^45^. Although the 190–200 nm region (π → π* transition) can provide additional structural detail, measurements in this range are often affected by strong absorbance of aqueous buffers, resulting in low light intensity and excessive noise at the photomultiplier tube^46^. To avoid these artifacts and maintain quantitative accuracy, the lower wavelength limit was set to 200 nm, consistent with standard practice in far-UV CD studies of proteins in physiological buffers^8,25,28,47,48^.

**Fig. 5:**
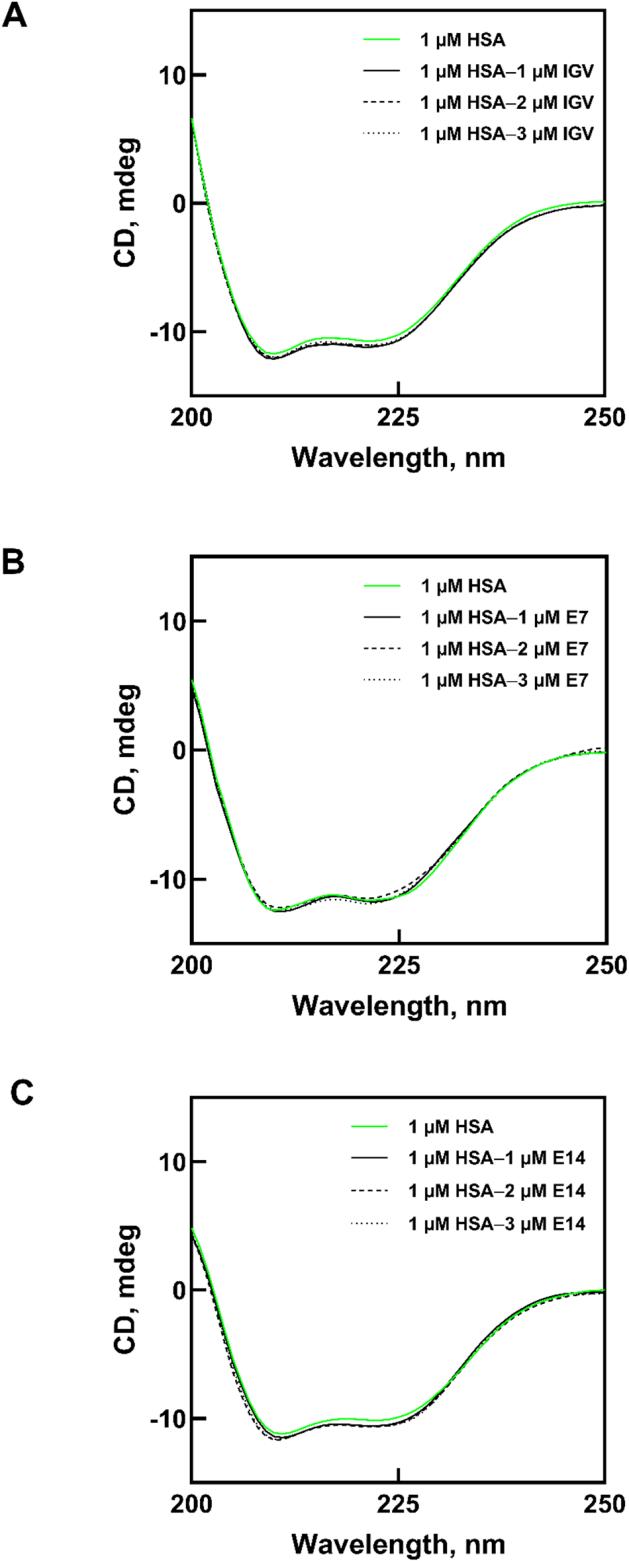
Far-UV CD spectra of HSA without and with the addition of varying concentrations of (**A**) IGV, (**B**) **E7** and (**C**) **E14**.

The far-UV CD spectra of HSA in the absence and presence of IGV, **E7**, and **E14** are shown in Fig. 5. The two negative peaks at 208 nm and 222 nm represent the predominantly helical structure of HSA^49^. The α-helix content of unbound HSA in this study was 63.8%, which is consistent with crystallographic data (~ 67%)^50,51^ and values reported in similar studies^25,52^. Importantly, these spectral features were maintained upon the addition of up to three molar excesses of the ligands. According to BeStSel predictions (Table 2), the presence of IGV only subtly affected the α-helical content; the β-sheet content remained exceptionally low (< 1%), while the β-turn composition was relative stable between 7.4% and 9.7%. This finding suggests that the presence of IGV had no significant effect on the secondary structure of HSA, consistent with the minimal structural perturbations typically observed upon binding of small ligands^53–55^.

Interestingly, the interaction of **E7** and **E14** with HSA resulted in similarly minor structural modifications, despite being strongly bound to the protein as demonstrated earlier. For both ligands, a small degree of increment in α-helical and β-sheet content was observed, with the β-turn percentage being consistent at ~ 9%. Hence, the conformational response to **E7/E14** binding also appears to be characterized by minimal perturbation of protein structural integrity. Additionally, the increased α-helical content may suggest a potential stabilizing effect of **E7** and **E14** on the protein structure. Ligand binding to proteins often stabilizes certain structural elements such as α-helices, aided by hydrogen bonding, electrostatic interactions, and hydrophobic interactions^56^. Consequently, HSA in the ligand-bound form may show a higher percentage of α-helices than in the free form.

**Table 2:** Secondary structure composition of HSA in the unbound state and in the presence of IGV, **E7**, and **E14** based on the bestsel analysis.

Compound	[HSA]: [Ligand]	α–Helix, %	β–Sheet, %	β–Turn, %	Others, %
HSA	-	(63.8 ± 2.8)	0	(9.6 ± 0.2)	(26.6 ± 3.9)
IGV	1:1	(60.2 ± 2.5)	(0.4 ± 0.1)	(9.7 ± 0.2)	(29.7 ± 2.8)
	1:2	(62.7 ± 1.9)	(0.6 ± 0.1)	(9.1 ± 1.6)	(27.6 ± 7.4)
	1:3	(64.7 ± 3.1)	(0.4 ± 2.5)	(7.4 ± 1.7)	(27.5 ± 7.4)
**E7**	1:1	(62.4 ± 2.6)	(3.2 ± 0.5)	(8.5 ± 0.6)	(25.9 ± 2.7)
	1:2	(64.2 ± 0.2)	(2.7 ± 0.5)	(9.1 ± 0.6)	(26.3 ± 2.8)
	1:3	(65.9 ± 1.2)	(3.8 ± 0.8)	(8.7 ± 0.9)	(21.7 ± 0.5)
**E14**	1:1	(66.1 ± 0.9)	(3.6 ± 0.4)	(9.0 ± 1.2)	(21.3 ± 1.8)
	1:2	(67.4 ± 0.8)	(1.7 ± 1.3)	(9.7 ± 0.5)	(24.0 ± 1.2)
	1:3	(69.9 ± 0.8)	(1.8 ± 0.8)	(9.2 ± 0.5)	(19.1 ± 0.9)

Near-UV CD spectroscopy measurements are often recorded between 250 and 350 nm, where the aromatic amino acids Trp, Tyr, and Phe exhibit characteristic absorption bands that provide information about the tertiary structure and environment of these residues in proteins. These residues are usually found in the interior of the protein and their local environment can undergo changes upon ligand binding^57^. Further, the near-UV CD spectrum of proteins is also influenced by the asymmetry of disulfide bridges, though not as strongly as the aromatic chromophores.

**Fig. 6:**
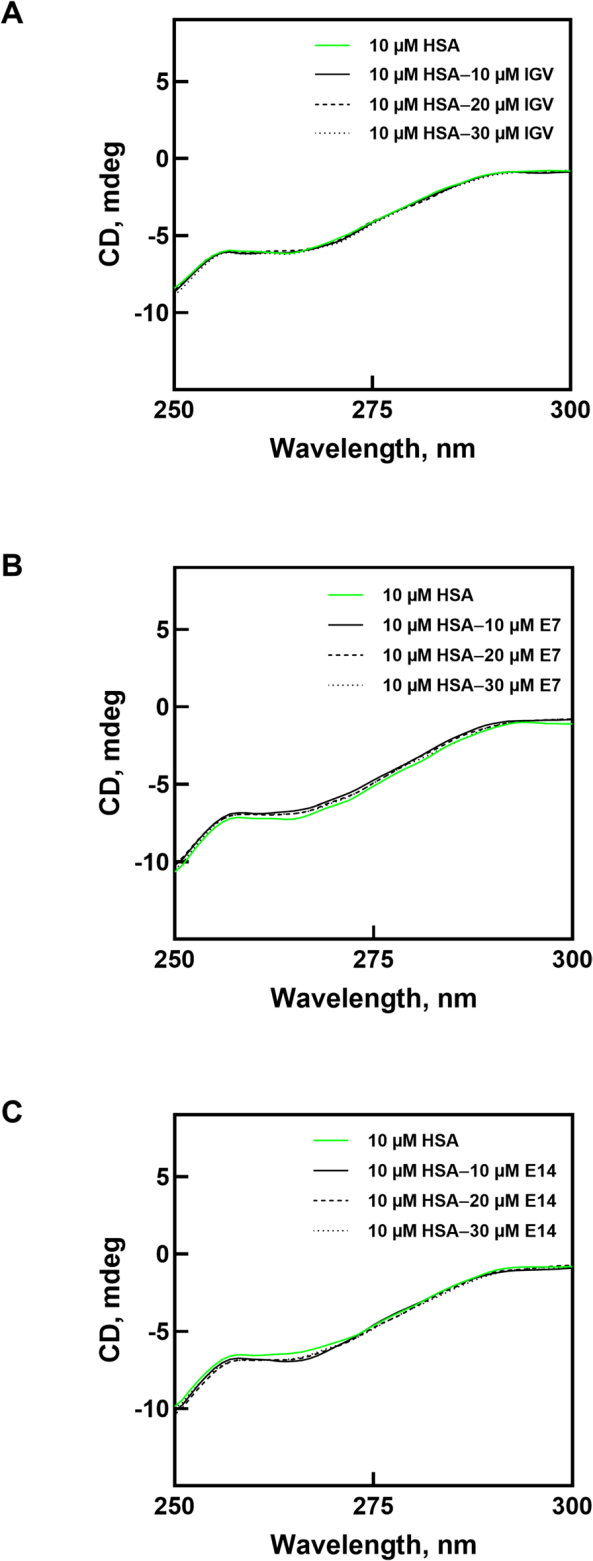
Near-UV CD spectra of HSA without and with the addition of varying concentrations of (**A**) IGV, (**B**) **E7** and (**C**) **E14**.

HSA contains one Trp residue, 18 Tyr, 31 Phe, and 17 pairs of disulfide bonds^58^. The combined chiral properties of these residues together contribute towards the overall near-UV CD profile of the protein (Fig. 6). The near-UV CD spectrum of HSA exhibits two negative peaks at around 262 nm and 269 nm that are contributed by Phe residues, while spectral features near 275 nm and 282 nm are attributable to Tyr residues^59^. Trp residues, particularly through the indole ring, contribute to the CD spectrum at 290–300 nm; while a broad negative tail, accompanied by a weak signal extending up to 300 nm, indicate the involvement of disulfide bonds^59^.

Possible changes in the tertiary structure of HSA after the addition of IGV, **E7**, and **E14** were analyzed through changes in the CD spectra. Figure 6A shows that the addition of IGV produced spectra that almost perfectly overlapped with each other, including that of the free HSA. Although the IGV: HSA molar ratio was increased to 3:1, no noticeable changes were observed, indicating that the tertiary structure of HSA was unaffected. On the other hand, the addition of **E7** and **E14** did lead to some gradual changes in the CD spectra, especially in the 260–270 nm range (Fig. 6B and C). Nevertheless, these changes are considered minor as the overall near-UV CD spectral profile was retained even upon binding at the highest ligand concentration. Therefore, it can be safely concluded that the binding of **E7** and **E14** also had no negative effect on the tertiary structure of HSA, and consequently, its biological function^60,61^.

### Molecular docking analysis

Based on the calculated pK_a_ values using ChemAxon Chemicalize (Fig. S5), the ligands exhibit distinct protonation states at physiological pH (7.4) that influence their binding interactions with HSA. MYR exists primarily as an anionic species, with its carboxylic acid group (pK_a_ 4.95) being 99.64% deprotonated. In contrast, the **E7** and **E14** compounds remain predominantly cationic, with their basic amine groups (pK_a_ 8.71) being 95.29% protonated under physiological conditions. Thus, molecular docking analysis on the interaction with HSA was carried out for the predominant protonation states of the ligands at physiological pH.

Using these defined protonation states, molecular docking studies were performed to complement the ITC experiments and to provide structural insights into the binding of MYR (as the reference ligand) and IGV derivatives to HSA. For each docking simulation, one hundred ligand conformations were generated and arranged in order of increasing binding energy. Only the conformations with the lowest binding energy were selected for further analysis with HSA, as these exhibit the highest affinity for the protein, reducing the possibility of dissociation and increasing the stability of the protein–ligand complex.

**Fig. 7:**
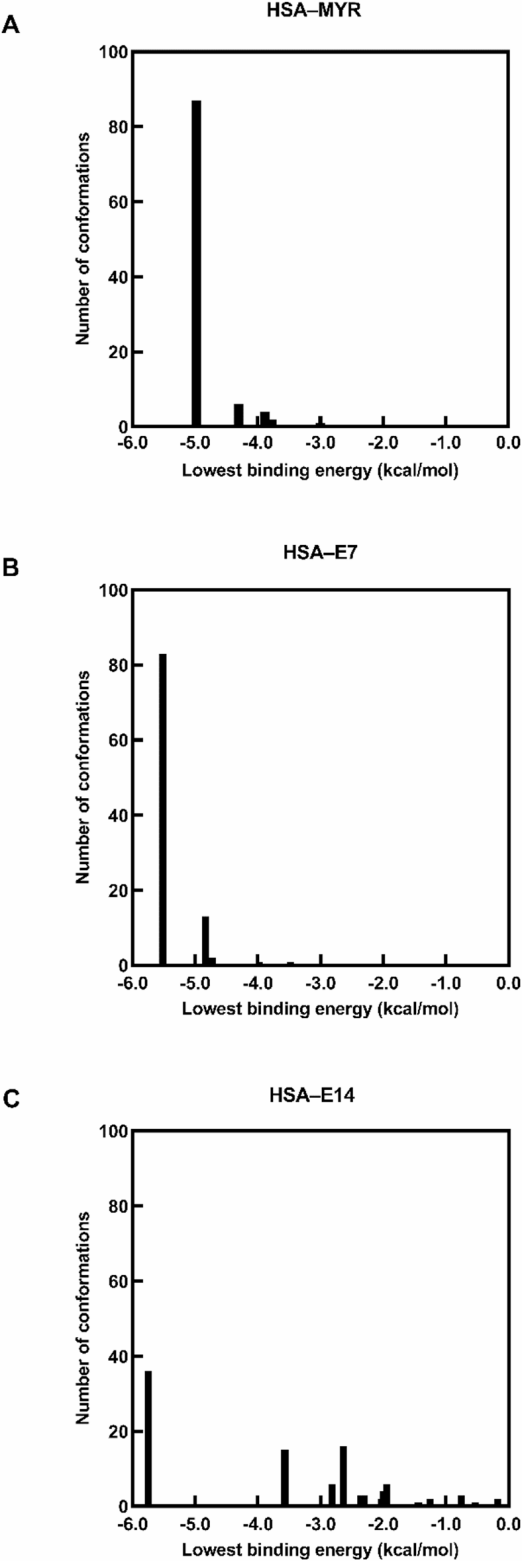
Analysis of distinct cluster conformations for the docking of (**A**) MYR, (**B**) **E7** and (**C**) **E14** to HSA.

The cluster conformational analysis of protein–ligand complexes revealed distinct binding patterns among the ligands. For the HSA–MYR complex, five conformational clusters were identified; however, the lowest binding energy clusters (–4.98 kcal/mol) accounted for 87% of the total conformers (Fig. 7A). This suggests that the binding of MYR to the FA5 site of HSA is highly specific (Fig. S6), as mediated primarily by hydrophobic interactions with the hydrocarbon tail of the molecule, supplemented by hydrogen bonding with the carboxylic head along with van der Waals forces. An attractive charge interaction was observed between HSA (Lys-524) and the carboxylate group (COO⁻) of MYR, forming part of the electrostatic interactions within the ligand–protein complex (Table 3).

**Fig. 8:**
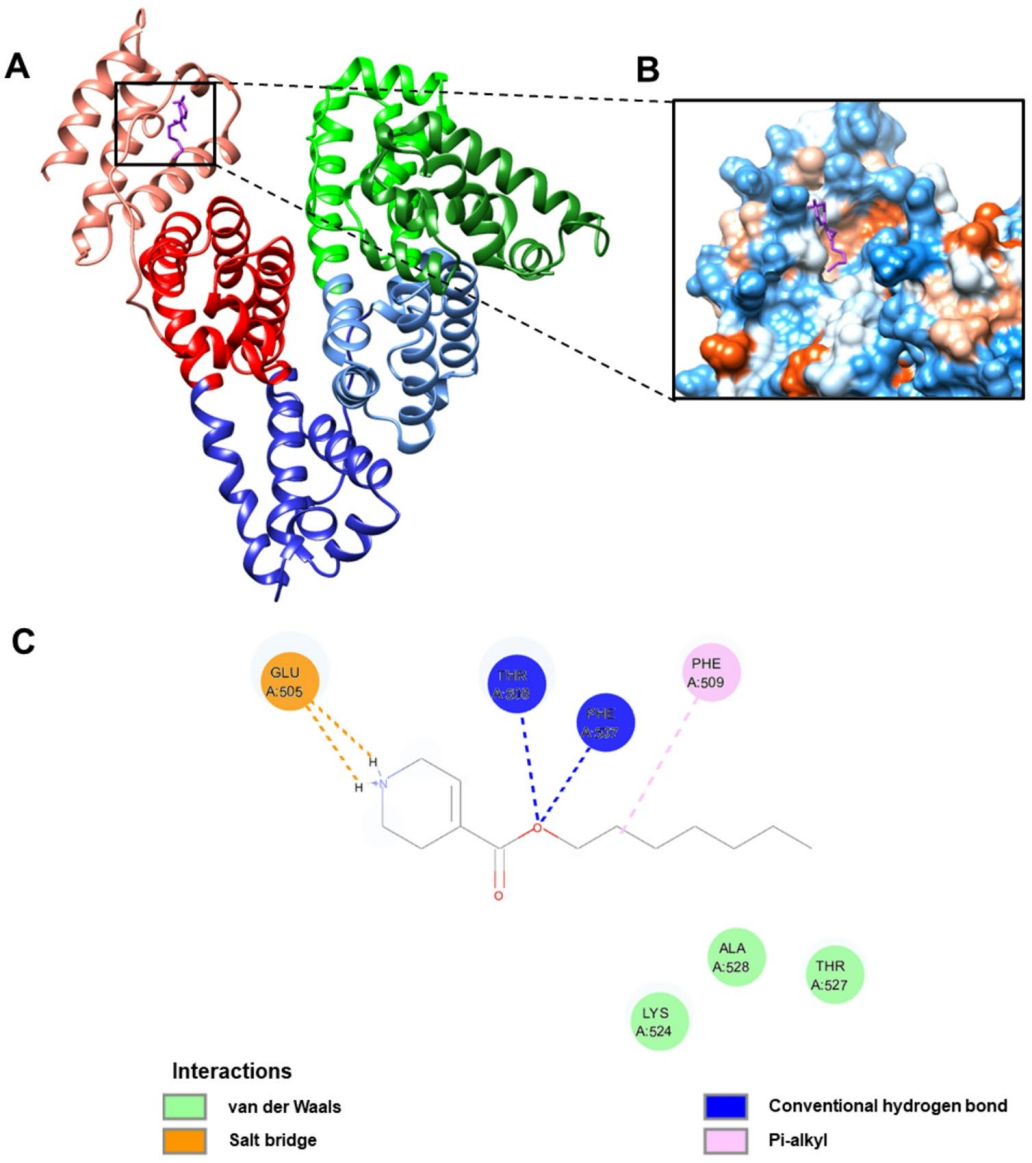
(**A**) Conformation with the lowest binding energy (–5.52 kcal/mol) of **E7** (purple) docked onto the FA5 site of HSA. (**B**) Zoomed view highlighting the predicted binding pocket for **E7**, shown through 3D surface model colored by hydrophobicity. (**C**) Schematic depiction of the various intermolecular interactions involved in the predicted HSA–**E7** binding mode.

**Fig. 9:**
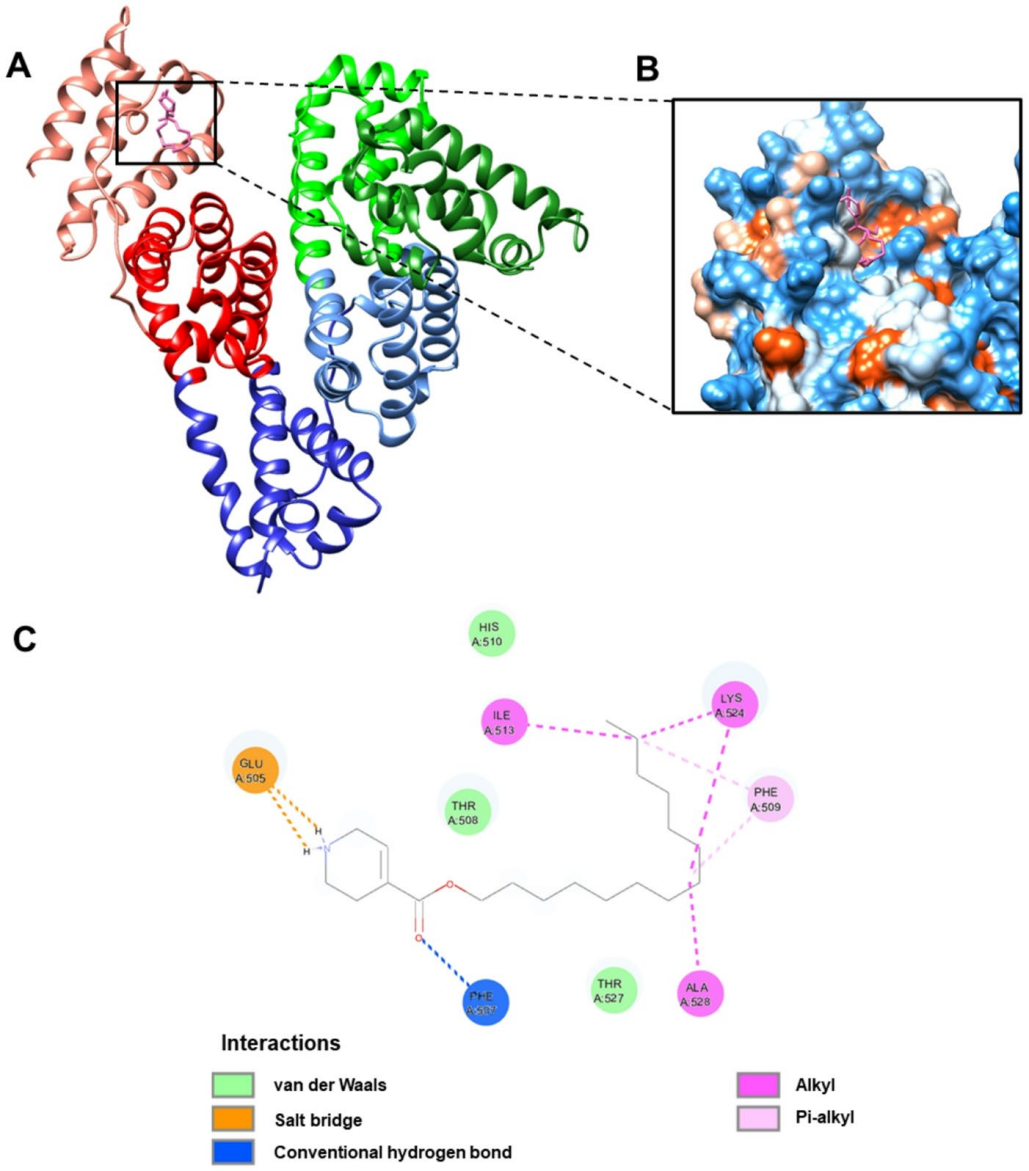
(**A**) Conformation with the lowest binding energy (–5.75 kcal/mol) of **E14** (pink) docked onto the FA5 site of HSA. (**B**) Zoomed view highlighting the predicted binding pocket for **E14**, shown through 3D surface model colored by hydrophobicity. (**C**) Schematic depiction of the various intermolecular interactions involved in the predicted HSA–**E14** binding mode.

As for the HSA–**E7/E14** complexes, a rather predictable binding pattern was observed, which resembled that of the HSA–MYR complex. The lowest binding energy cluster (–5.52 kcal/mol) of **E7**, also representing the most populated configuration, contained 83 out of the total 100 conformers (Fig. 7B). The presence of this dominant cluster indicates that the binding of **E7** to HSA demonstrates high specificity. The binding specificity was mediated through a combination of conventional hydrogen bonds, alkyl hydrophobic interactions, salt-bridge, and stabilizing van der Waals forces (Fig. 8; Table 3). Similarly, while the HSA–**E14** complex exhibited thirteen conformational clusters, the lowest binding energy cluster (–5.75 kcal/mol) was predominant, comprising 36% of conformers (Fig. 7C). Likewise, the protein–ligand association was facilitated by an extensive network of interactions including conventional hydrogen bonds, salt-bridge, pi-sigma hydrophobic interactions, alkyl interactions, along with van der Waals contacts (Fig. 9; Table 3).

The shared characteristic of specific and energetically favorable interactions among MYR, **E7**, and **E14** with HSA is expected due to their structural similarity of possessing an aliphatic chain. Further, the albumin binding affinity of the ligands correlated with the increase in hydrophobicity from **E7** to **E14**. The introduction of the non-polar moieties via esterification enhanced hydrophobic interactions with HSA, as evidenced by the extensive alkyl and pi-sigma interactions observed with **E7** and **E14**, contributing to their higher binding affinity.

**Fig. 10:**
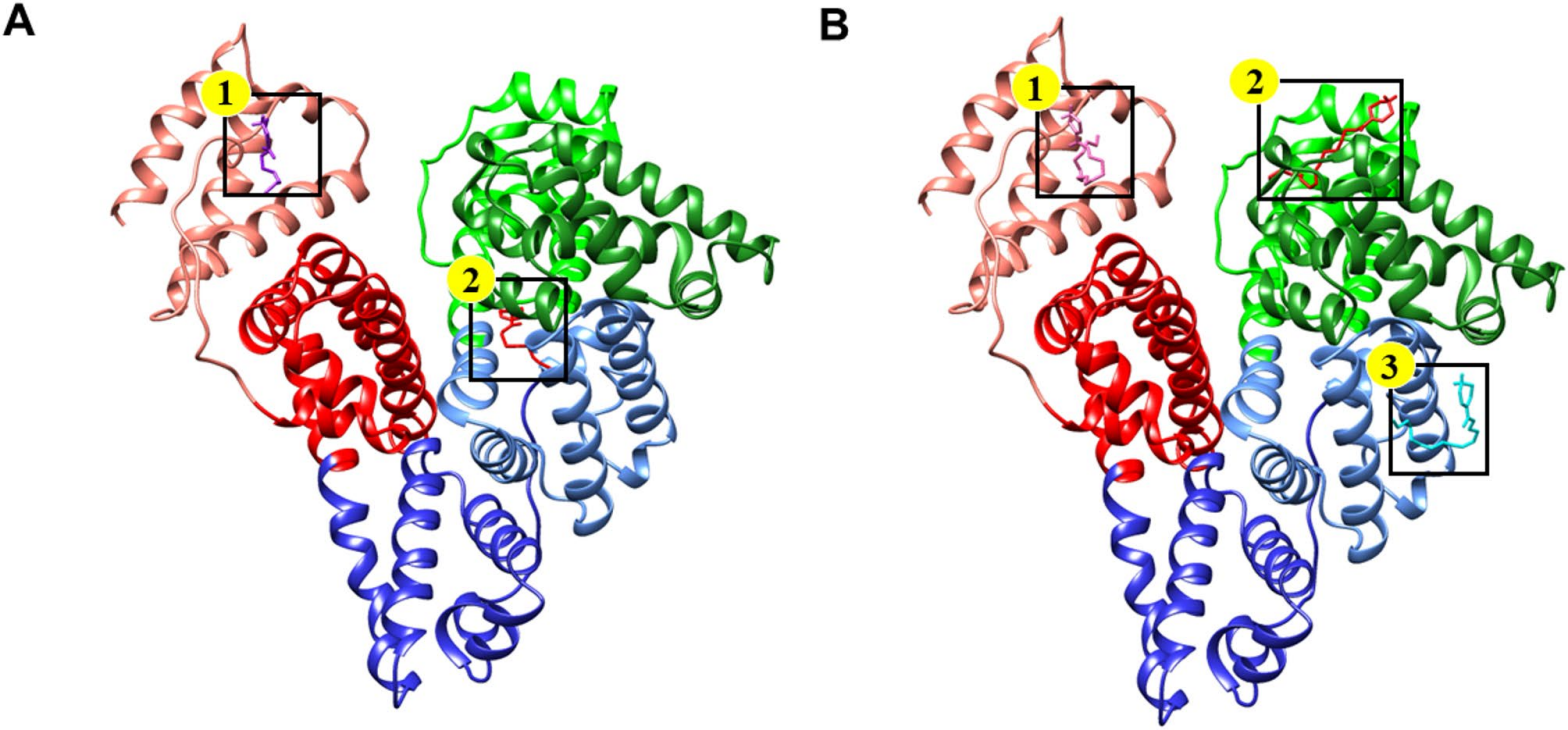
Blind docking results illustrating the multiple binding sites of (**A**) **E7** and (**B**) **E14** on HSA.

Although the docking results indicated well-defined binding modes, ITC analysis implies the presence of multiple binding sites for both **E7** and **E14**, suggesting complex interaction behavior with HSA. Hence, to explore other potential interaction regions, blind docking simulations were performed. For **E7**, an additional binding site at the interface between subdomains IB and IIA was identified, corresponding to the FA2 region (Fig. 10A). This site functions as a secondary recognition cleft that accommodates carboxylate head groups and hydrophobic tails^62^. As for **E14**, blind docking revealed two additional binding sites (Fig. 10B). One was located between subdomains IA and IIA, positioned near Sudlow’s site II (FA3 and FA4), which is known for its stereoselectivity and preference for aromatic carboxylates^62,63^, while the other was identified within subdomain IB, corresponding to the FA1 region—a major drug-binding site that overlaps with the heme-binding pocket and is characterized by π–π stacking interactions^59^. The presence of these additional binding modes is consistent with the binding stoichiometry observed in ITC, supporting the conclusion that both **E7** and **E14** could occupy multiple binding pockets on HSA.

**Table 3:** Interaction profiles of HSA with MYR, **E7** and **E14** at the FA5 binding site.

Ligand	Binding energy (kcal/mol)	Type of interaction	Bond type	Interacting residues
MYR	–4.98	Hydrogen bond	Carbon	Lys-524
		Attractive charge		Lys-524
		Hydrophobic interaction	Alkyl	Lys-524, Ala-528
			Pi-Alkyl	Phe-509
		van der Waals forces		Phe-507, Ile-513, Thr-527
**E7**	–5.52	Hydrogen bond	Conventional	Phe-507, Thr-508
		Salt bridge		Glu-505
		Hydrophobic interaction	Pi-alkyl	Phe-509
		van der Waals forces		Lys-524, Thr-527, Ala-528
**E14**	–5.75	Hydrogen bond	Conventional	Phe-507
		Salt bridge		Glu-505
		Hydrophobic interaction	Alkyl	Ile-513, Lys-524, Ala-528
			Pi-alkyl	Phe-509
		van der Waals forces		Thr-508, His-510, Thr-527

### Molecular dynamics simulations

Following the docking study, the optimal conformation for each protein–ligand complex was selected for molecular dynamics (MD) simulations, which involve studying the movements and interactions of atoms and molecules over time to provide insights into conformational changes, interactions, and responses to external factors. In this study, calculations such as root mean square deviation (RMSD), root mean square fluctuation (RMSF), radius of gyration (Rg), and hydrogen bond analysis were performed to determine the structural stability and flexibility of the protein upon ligand binding.

**Fig. 11:**
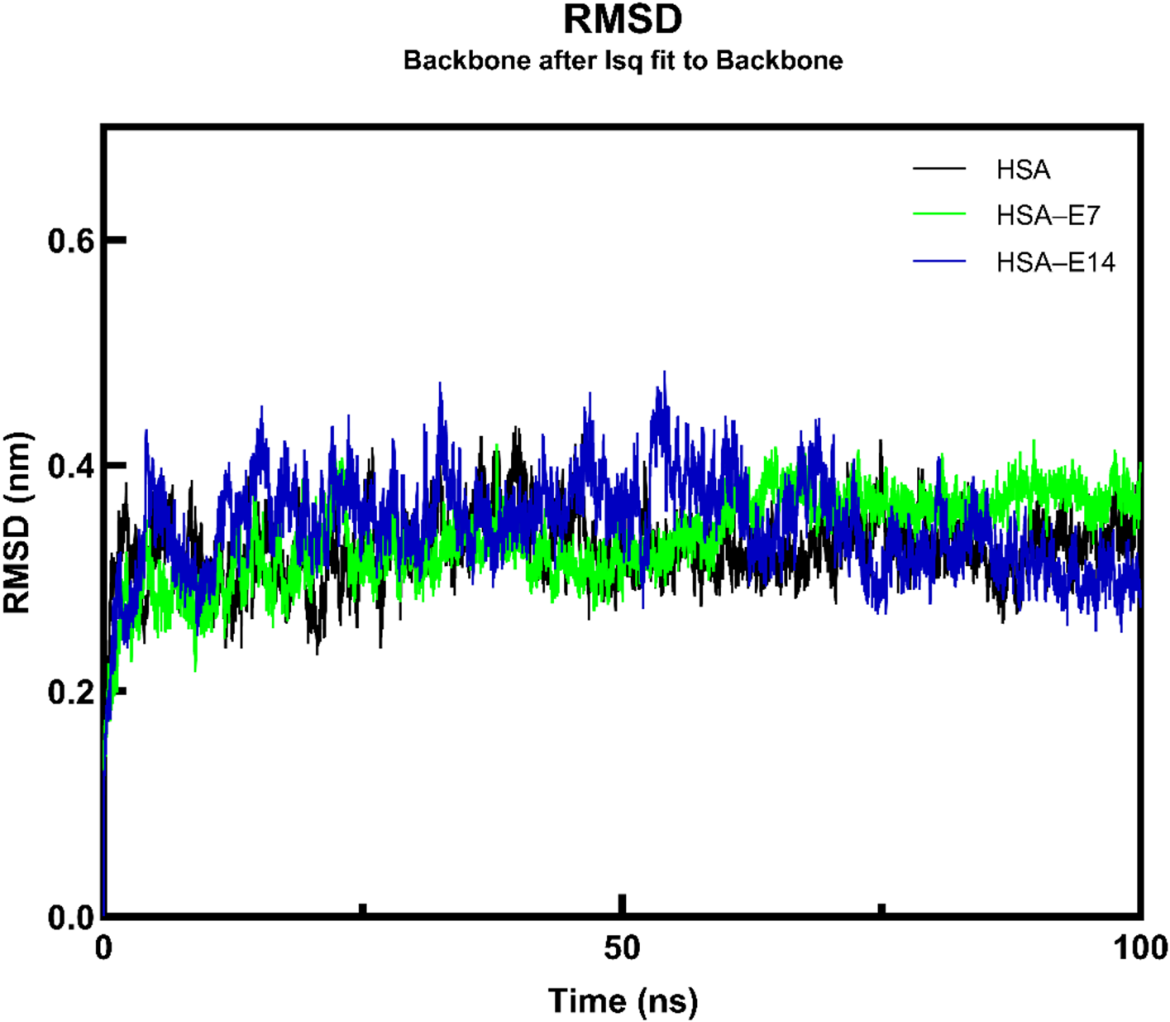
The RMSD of the backbone structure of HSA after least squares alignment in the absence and presence of **E7** and **E14**.

The initial analysis focused on RMSD, which quantifies the deviation of the protein backbone from its initial structure compared to its final conformation. The replicate results for HSA in the absence and presence of ligands demonstrated that the three trajectories exhibited consistent and stable patterns (Fig. S7). For each system, the most stable trajectory among the triplicates was selected for comparative analysis in Fig. 11. For the unbound HSA, the RMSD value increased from 0.16 nm to 0.34 nm at 50 ns, and stabilized at an average of 0.33 nm, indicating minor structural adjustments before reaching a stable conformation. On the other hand, both the HSA–**E7** and HSA–**E14** complexes recorded RMSD values of 0.37 nm and 0.34 nm, respectively, at the 100 ns mark. It is noteworthy that the protein, in the presence of **E14**, exhibited a RMSD value close to that of the free protein, indicating the stabilizing effect of the ligand on the structural integrity of HSA.

**Fig. 12:**
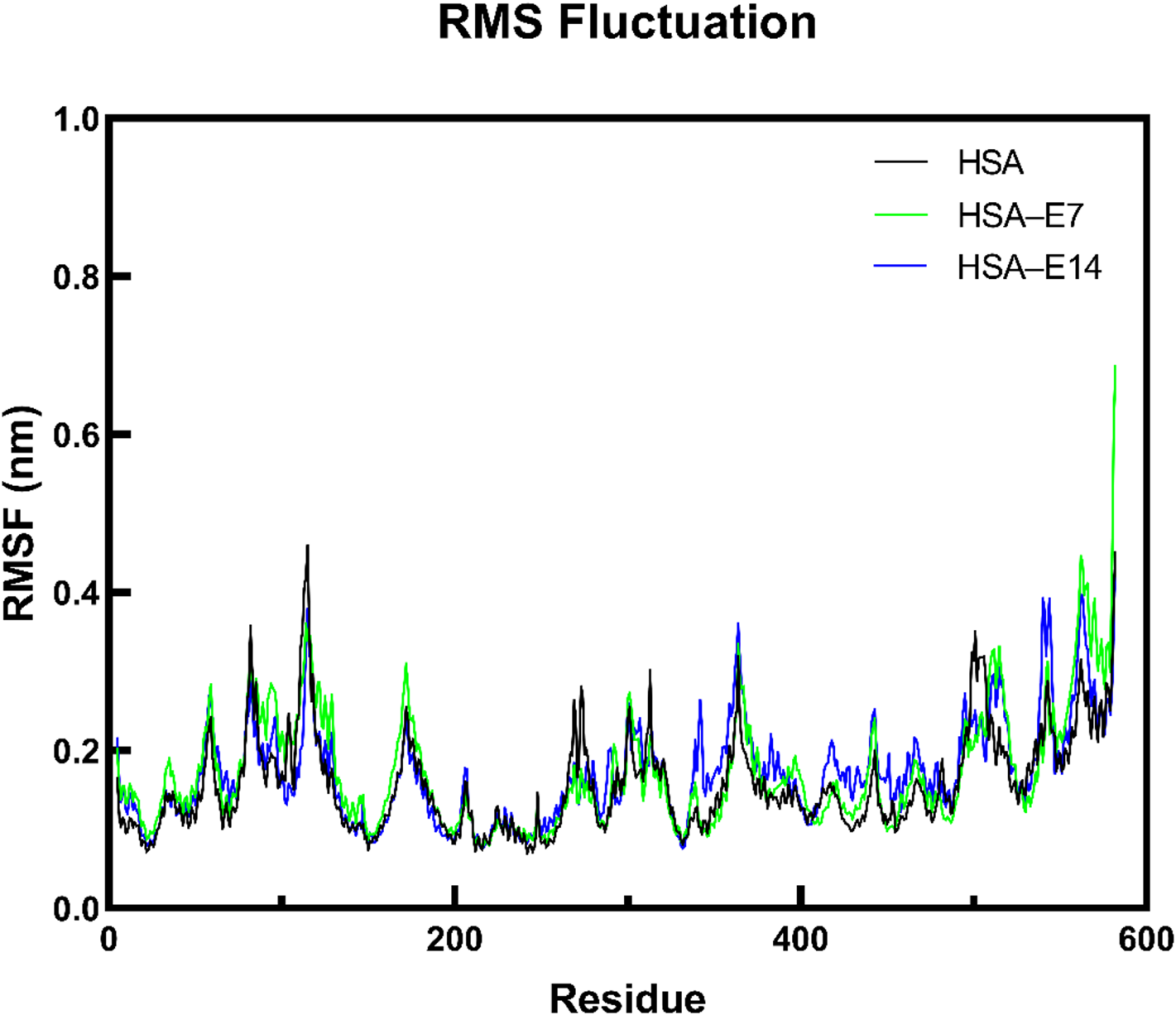
RMSF plots of HSA residues in the absence and presence of **E7** and **E14**.

To further understand the structural dynamics of the protein, RMSF analysis was performed to reveal regions showing significant conformational fluctuations upon interaction with the ligands. Notably, the replicate trajectories displayed consistent RMSF patterns (Fig. S8), with the most stable trajectory from each system selected for comparative analysis in Fig. 12. Compared with unbound HSA, the RMSF data (Fig. 12) showed that the presence of **E7** and **E14** led to an increase in average RMSF values, indicating greater flexibility at select residues to accommodate the ligands at the binding sites. In particular, major fluctuations were observed involving residues 109–112, 495–505, and 557–564 within the FA5 binding site of HSA, which are located in naturally flexible loop structures^64^.

**Fig. 13:**
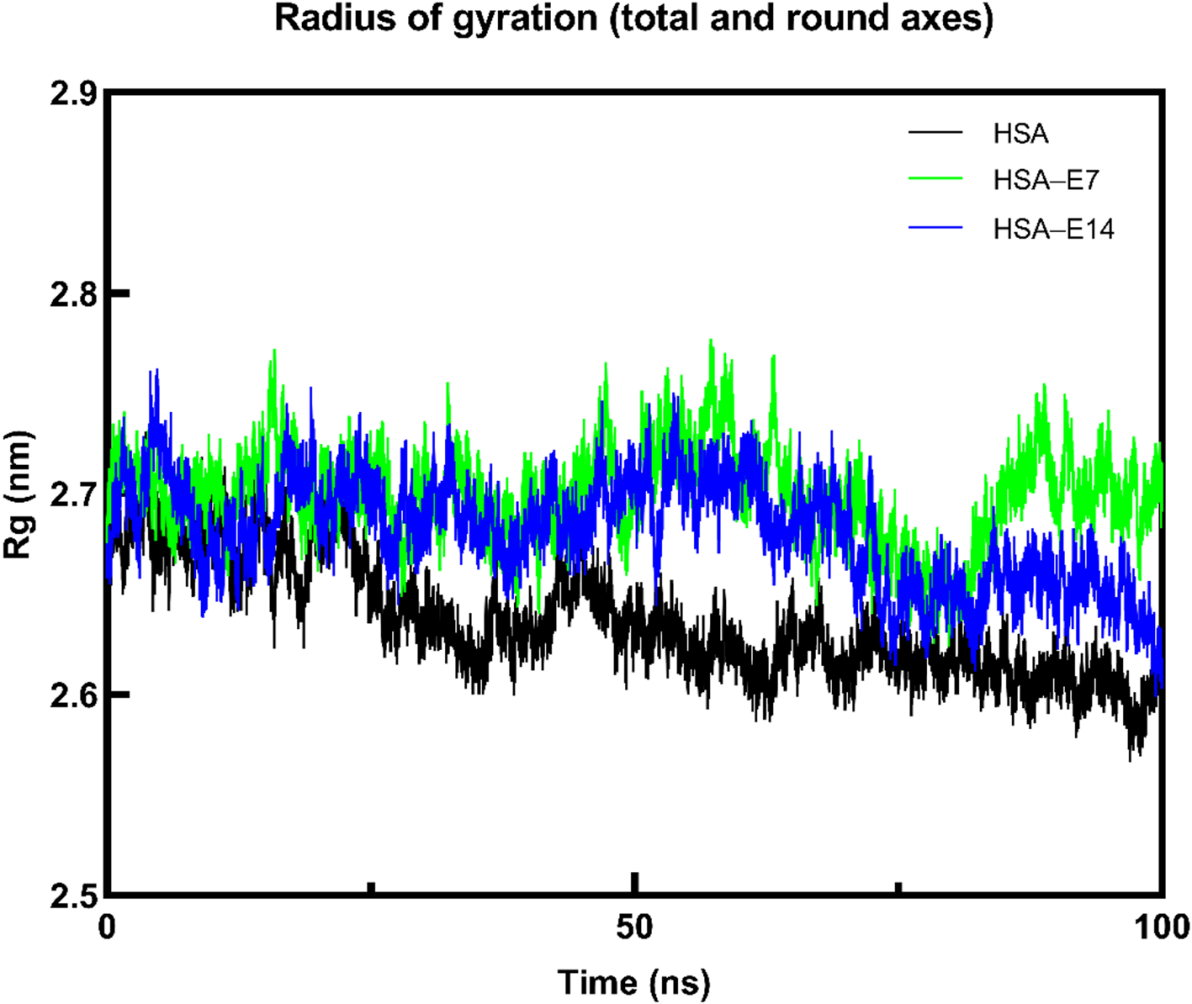
Evolution of Rg over time of HSA without and with the presence of **E7** and **E14**.

Complementing the RMSD and RMSF analyses, the radius of gyration (Rg) of HSA was also monitored to assess changes in protein compactness following ligand binding (Fig. 13). The average Rg value for the unbound HSA was 2.63 nm, while that of the complexes with **E7** and **E14** were 2.69 nm, and 2.68 nm, respectively. The higher Rg values of HSA upon association with **E7** and **E14** can be explained by the slight expansion of its size to fit the ligands into the binding site. Nevertheless, the minimal differences between Rg values (ranging within ~ 0.06 nm) suggest that all complexes maintained similar levels of compactness without considerable alteration of the overall protein conformation.

**Fig. 14:**
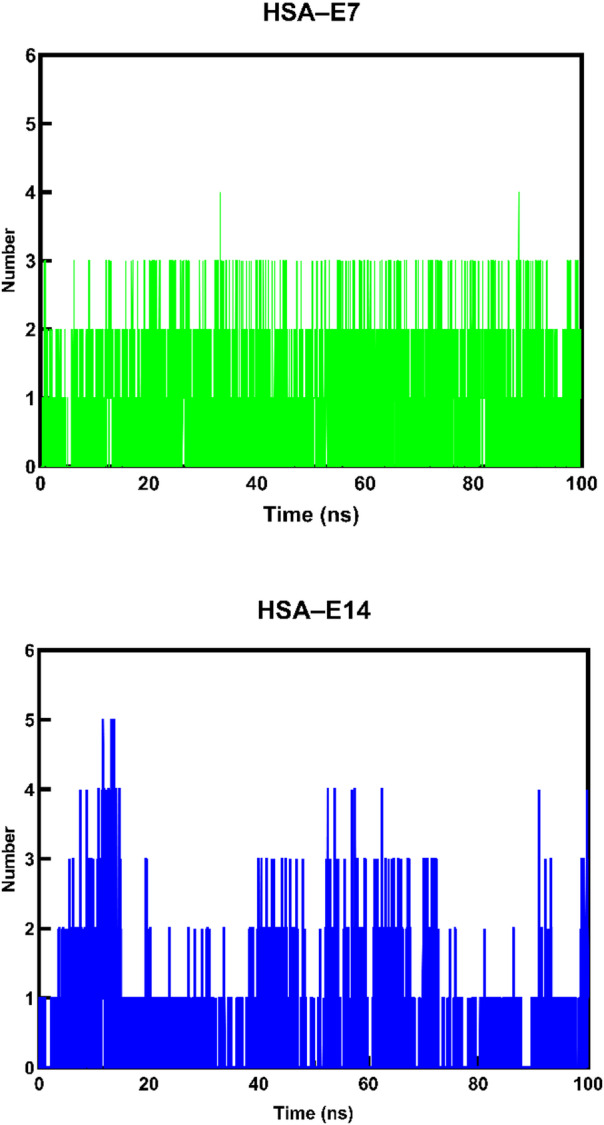
Number of predicted hydrogen bonds for interactions of HSA with **E7** and **E14**.

Finally, hydrogen bond analysis was conducted to understand the importance of hydrogen bond formation between HSA and the ligands during complexation. As shown in Fig. 14, the HSA–**E7** and HSA–**E14** complexes displayed averages of two and three hydrogen bonds, respectively. Both complexes demonstrated consistent hydrogen bonding throughout the simulation, indicating its significance in facilitating ligand binding. This observation generally aligns with the docking simulations and suggests the HSA–**E14** complex as the most stable.

## Conclusion

Biophysical and computational analyses revealed distinct binding patterns between HSA and IGV derivatives. UV-Vis spectroscopic studies confirmed the formation of protein–ligand complexes, while ITC measurements demonstrated significantly enhanced binding affinities for both IGV derivatives towards HSA, with **E14** exhibiting superior binding characteristics compared to **E7**. The increased hydrophobicity of the ester derivatives, particularly **E14**, facilitated stronger interactions with the hydrophobic pocket of HSA surface. CD spectroscopy confirmed that interactions with **E7** and **E14** preserved the secondary and tertiary structures of HSA, maintaining biological functionality of protein. Molecular docking studies corroborated the experimental findings, with the **E14**–HSA complex displaying the highest binding affinity, as supported by MD simulations demonstrating its superior complex stability. Molecular interactions between the ligands and HSA were mediated through hydrogen bonding and van der Waals forces, with hydrophobic interactions having greater significance for the ester derivatives. These findings provide valuable insights into the structure-binding relationships of IGV derivatives with HSA, offering useful information for rational drug design strategies. Nevertheless, the antiseizure effects of **E7** and **E14** were not directly assessed in this study, which focused on protein–ligand interactions and was limited by time, resources, and the ethical requirements of more complex pharmacological testing. Future studies incorporating in vivo models, alongside further structural investigations of **E14**, could advance the optimization of drug candidates with desirable binding and therapeutic properties.

## Supplementary Information

Below is the link to the electronic supplementary material.

Supplementary Material 1

## Data Availability

Data is provided within the manuscript and supplementary information files.
