# Supplementary material for: Improved albumin binding properties of Isoguvacine upon esterification as characterized by biophysical and computational tools
Source: Sci Rep. 2025 Nov 25;15:41973. doi: 10.1038/s41598-025-25957-7 (PMC12647779; doi:10.1038/s41598-025-25957-7)
Supplement: Supplementary file 1 — Supplementary Material 1 [file 41598_2025_25957_MOESM1_ESM.docx]

**Supplementary Materials**

**
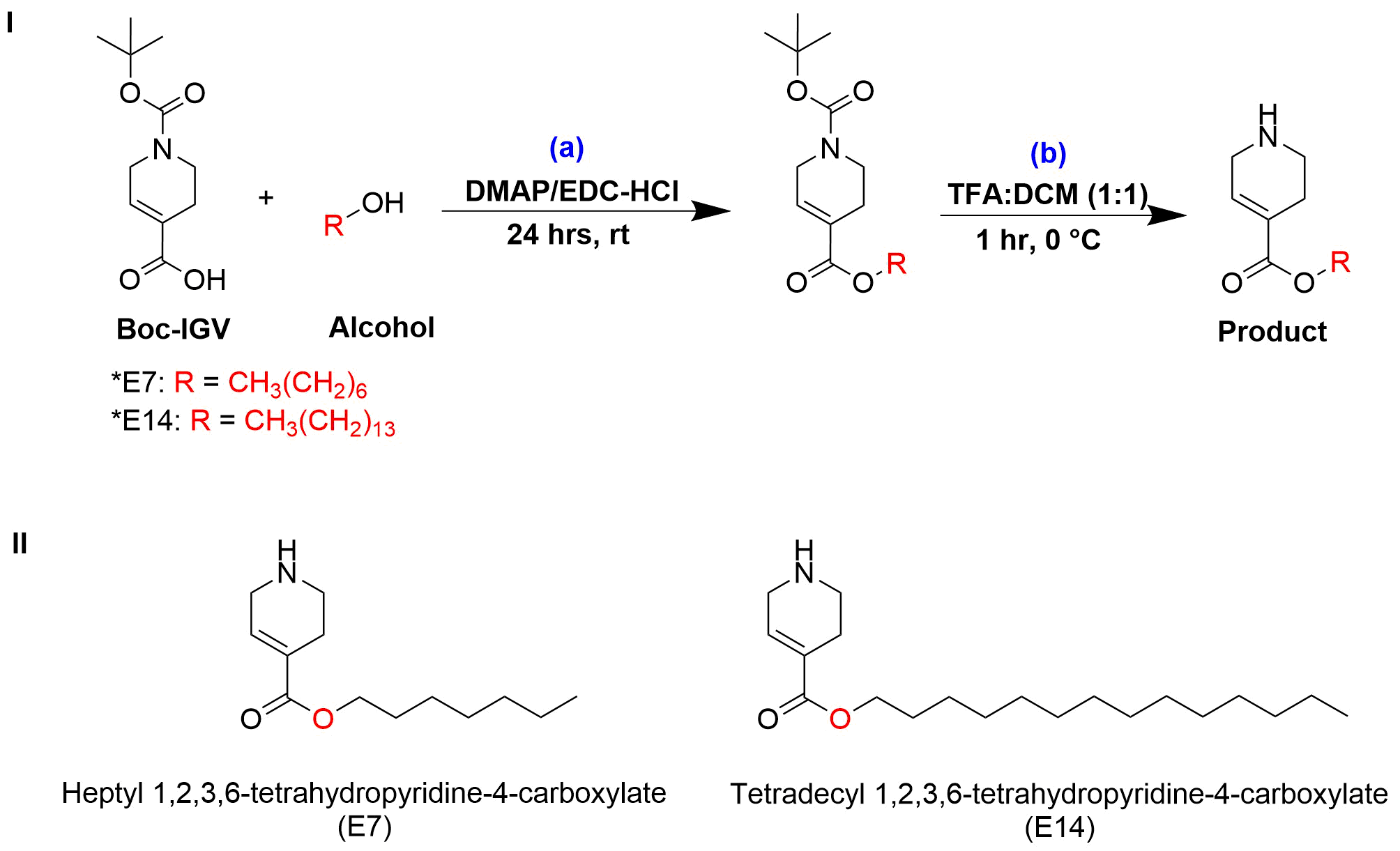
**

**Figure S1.** Synthesis of the IGV ester derivatives **E7** and **E14**. (a) Steglich esterification of Boc-protected isoguvacine (Boc-IGV) with heptanol or tetradecanol, catalyzed by DMAP/EDC·HCl (24 h, rt). (b) Deprotection using TFA/DCM (1:1, 0 °C, 1 h) to yield the final products—**E7** and **E14**.


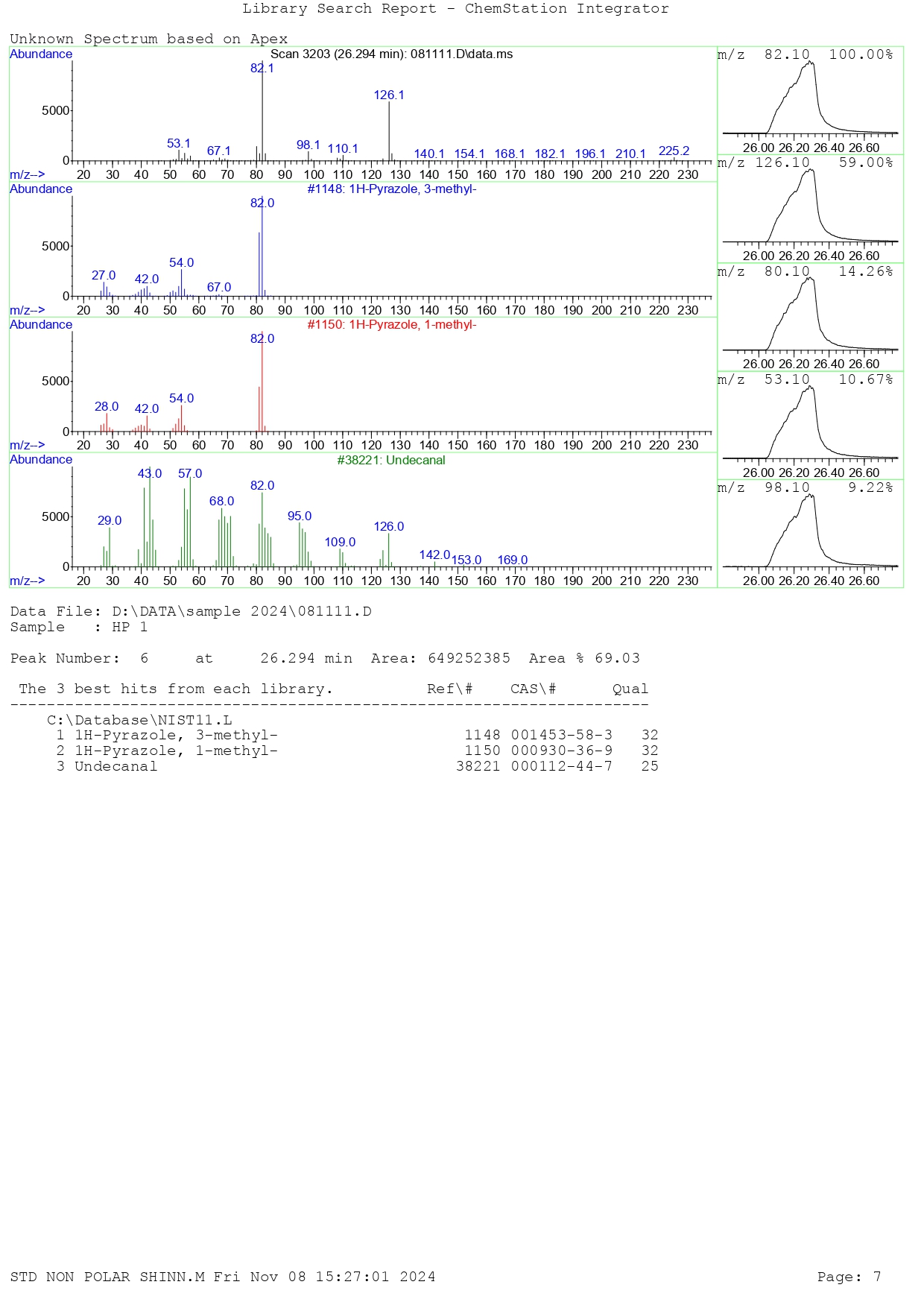


**A**


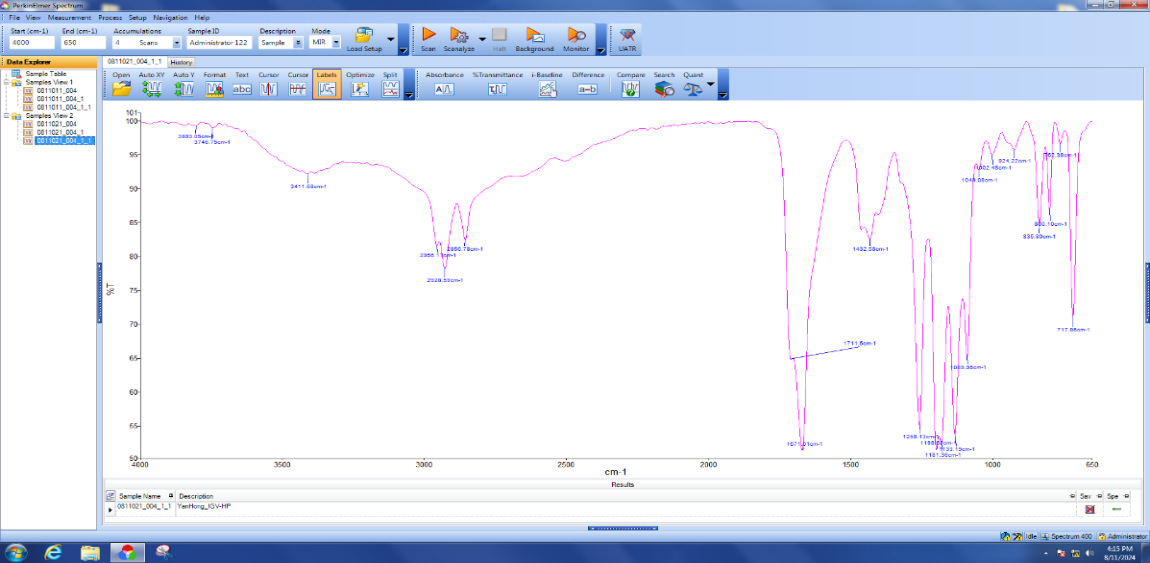


**B**


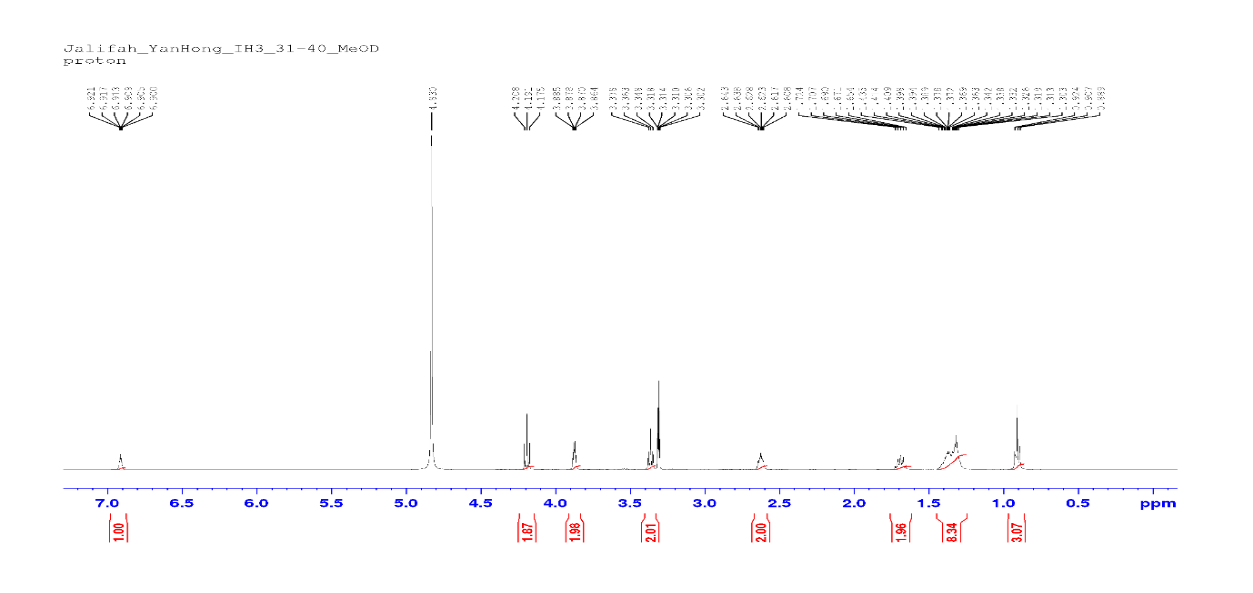


**C**


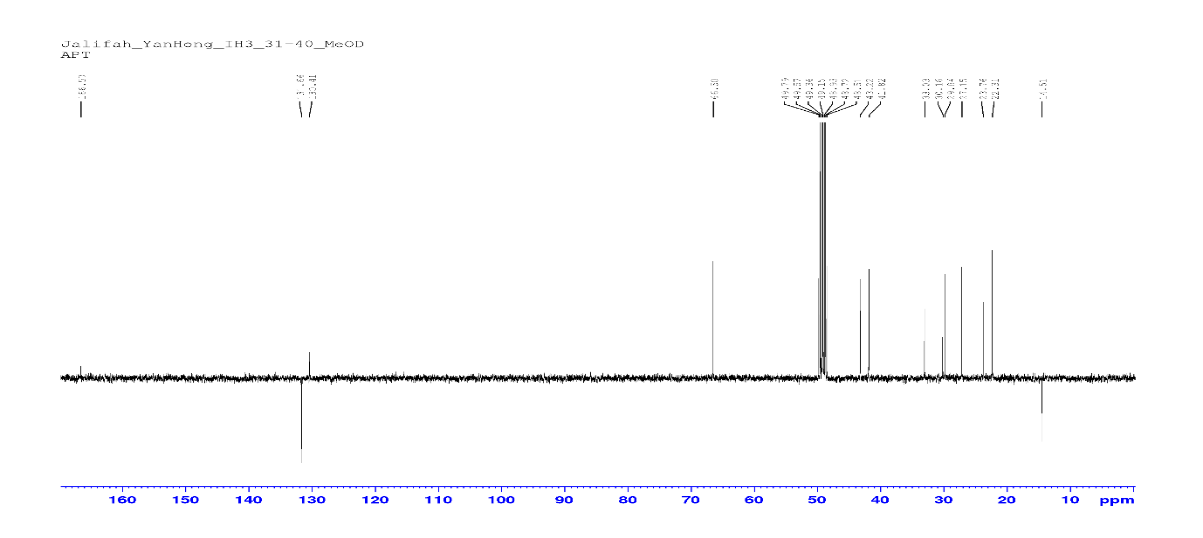


**D**

**Figure S2.** (A) GC-MS spectrum showing a molecular ion peak at *m/z* 225.2, consistent with the expected molecular weight of **E7**. (B) FT-IR spectrum of **E7** showing a strong absorption band at 1711.50 cm⁻¹ corresponding to the C=O stretching of the ester group. (C) ^1^H NMR spectrum (400 MHz, CD_3_OD) of **E7**, displaying signals attributed to aromatic, methylene, and aliphatic protons. (D) ^13^C NMR spectrum (100 MHz, CD_3_OD) of **E7**, showing signals for ester carbonyl, aromatic, and aliphatic carbon atoms.


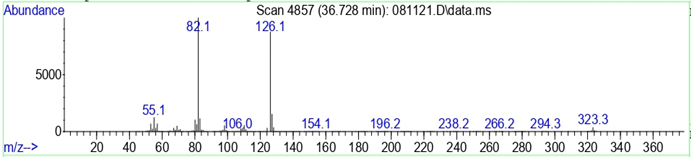


**A**


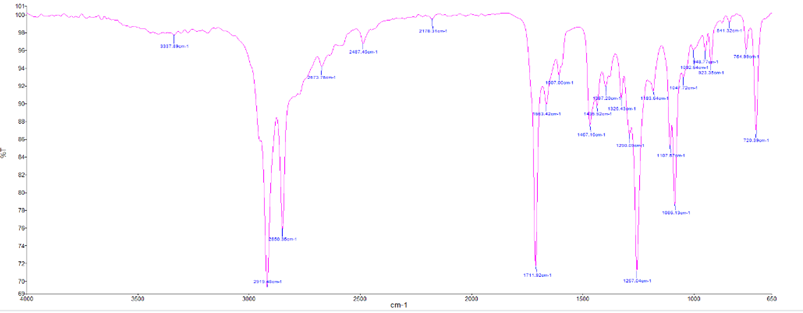


**B**


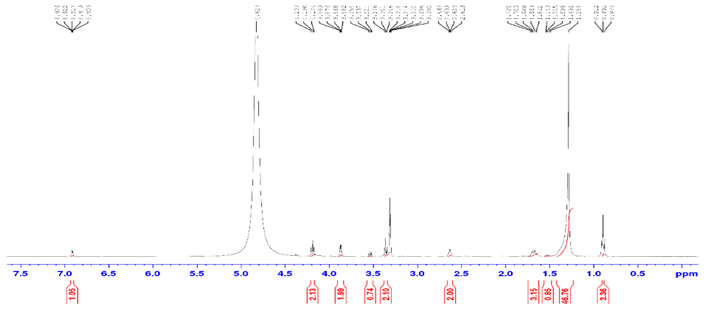


**C**


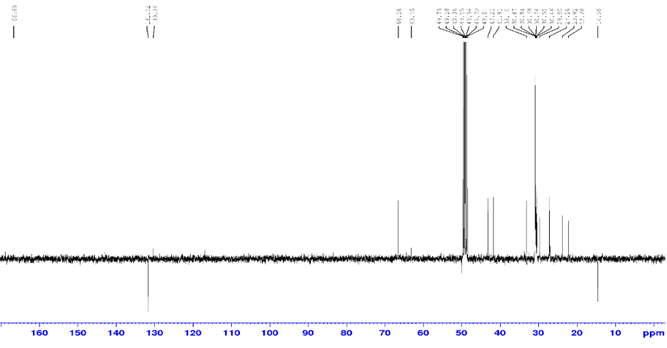


**D**

**Figure S3.** (A) GC-MS spectrum showing a molecular ion peak at *m/z* 323.3, consistent with the expected molecular weight of **E14**. (B) FT-IR spectrum of **E14** displaying a strong absorption band at 1711.92 cm⁻¹, attributed to the C=O stretching vibration of the ester group. (C) ^1^H NMR spectrum (400 MHz, CD_3_OD) of **E14**, displaying signals attributed to aromatic, ester-linked methylene, and long-chain aliphatic protons. (D) ^13^C NMR spectrum (100 MHz, CD_3_OD) of **E14**, showing resonances for ester carbonyl, aromatic, heteroatom-adjacent methylene, and aliphatic carbon atoms.


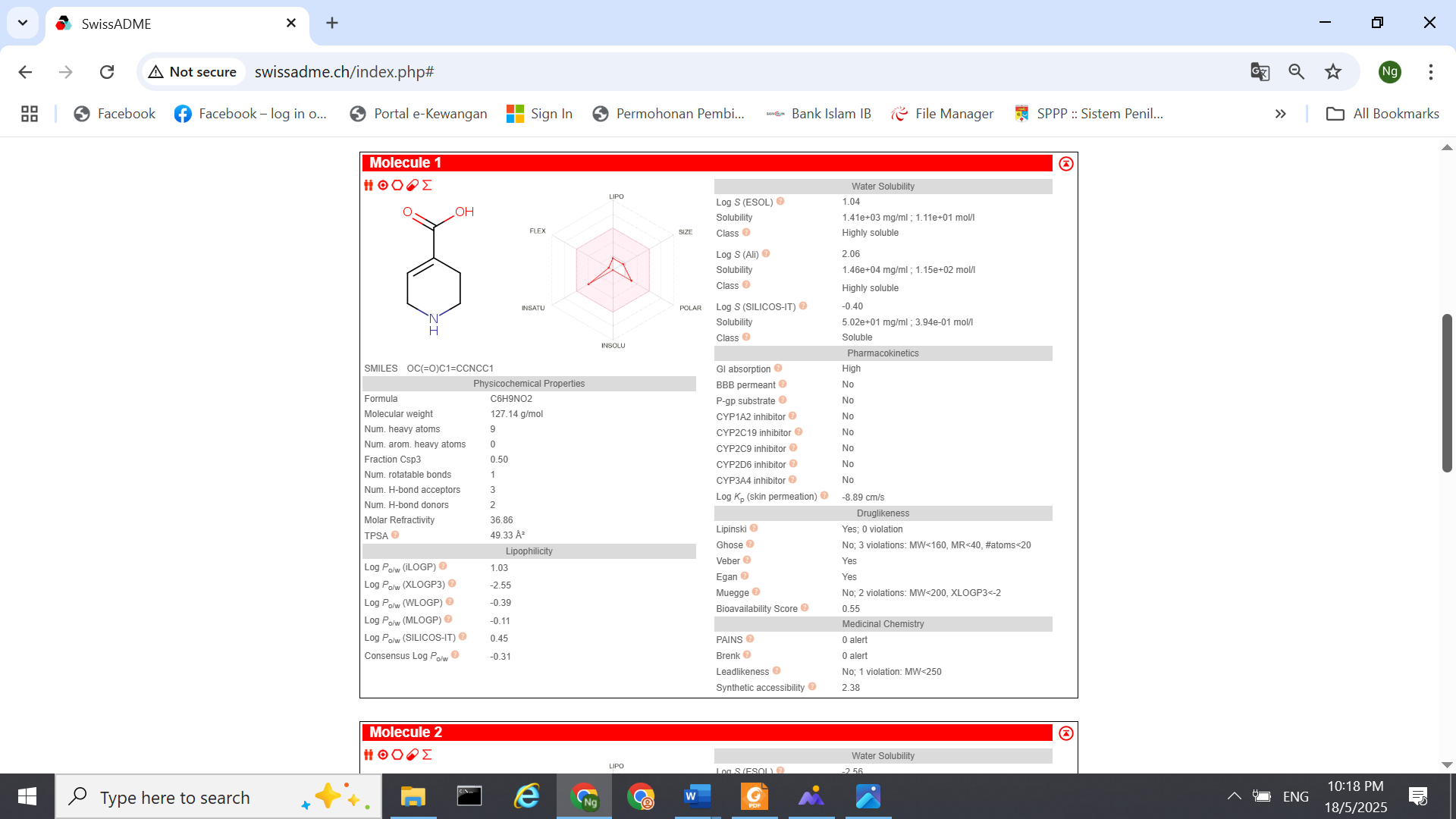


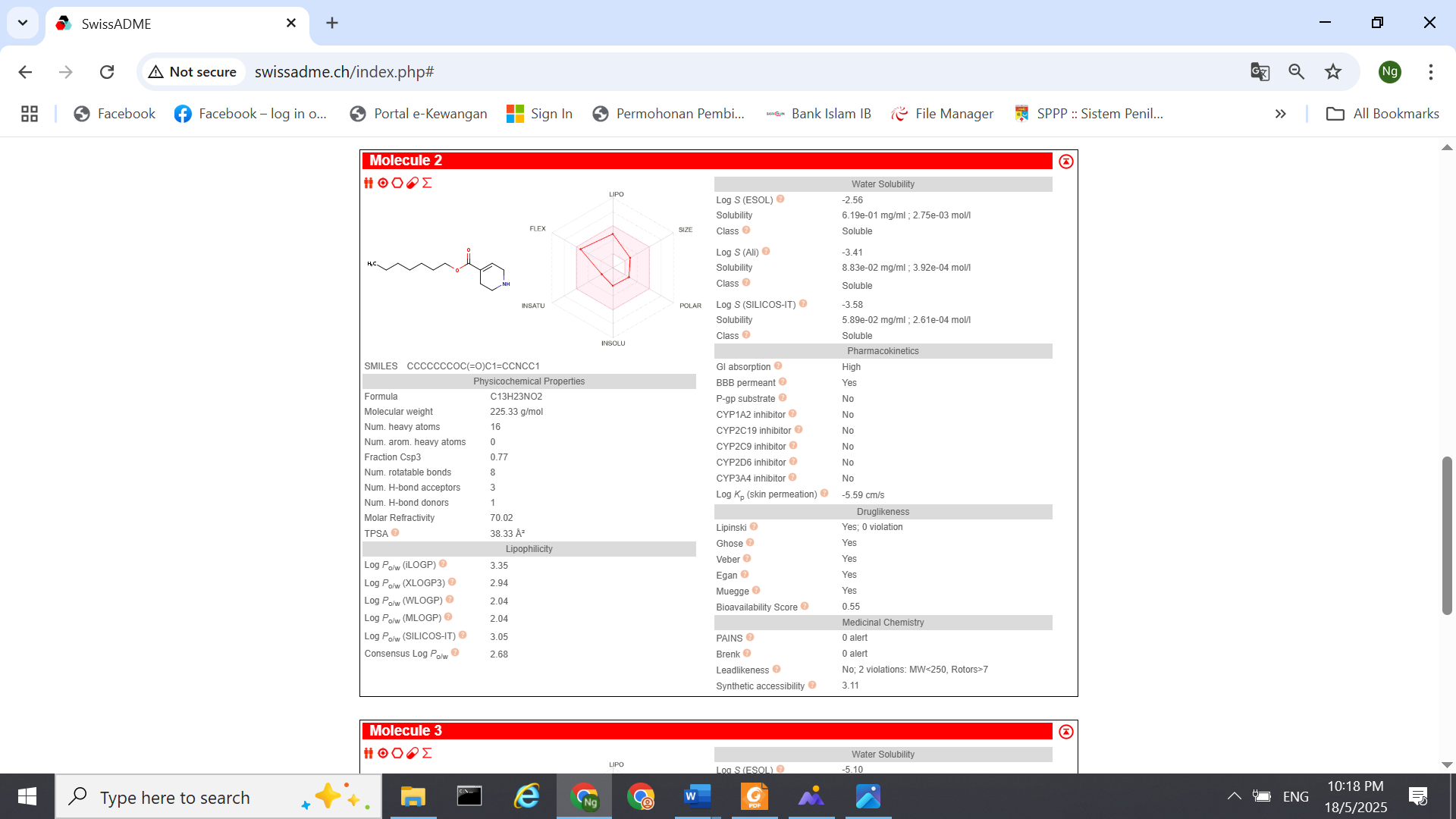


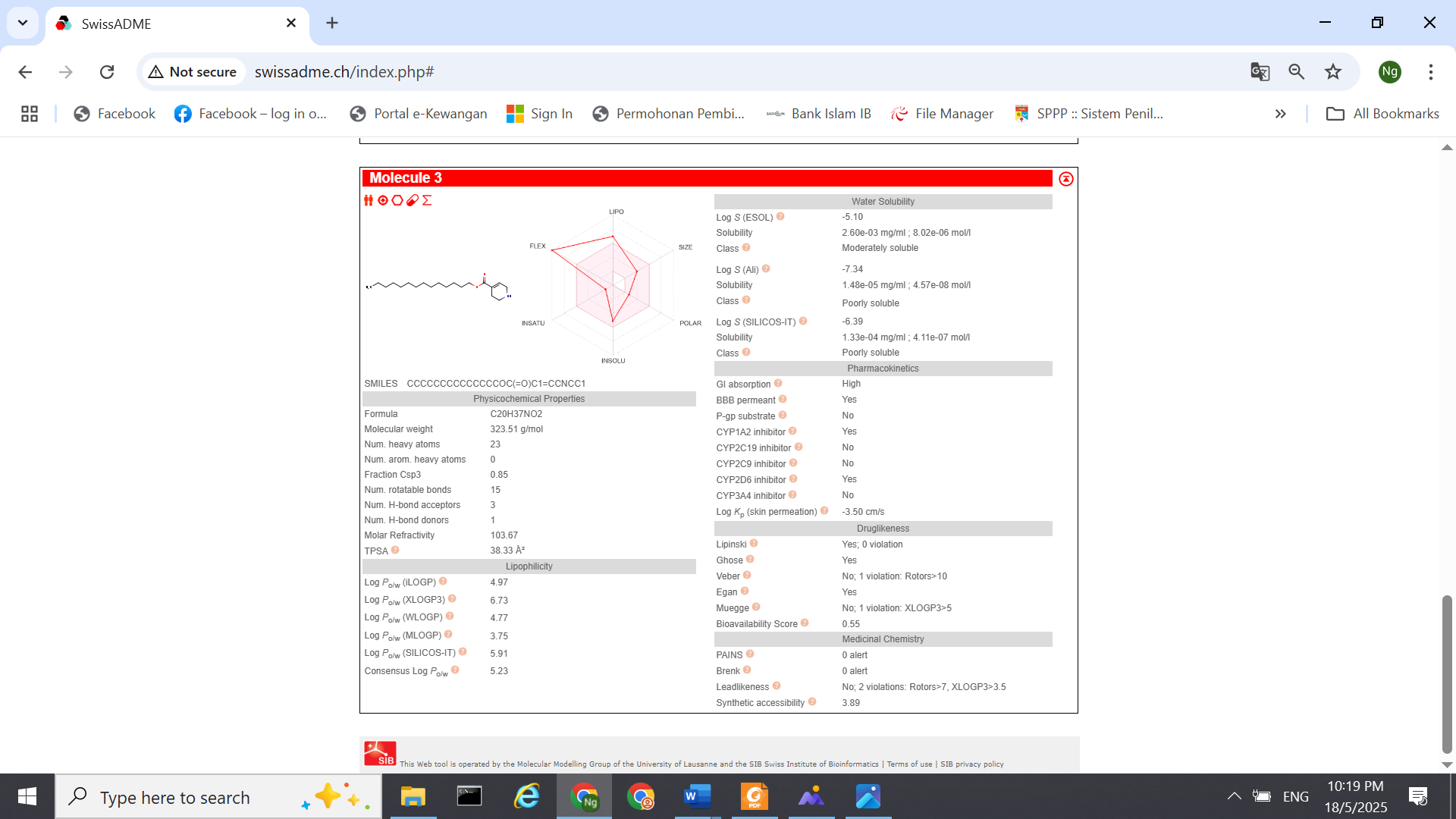


**Figure S4.** ADMET analysis using SwissADME predicts the blood-brain barrier permeability of IGV, **E7** and **E14.**


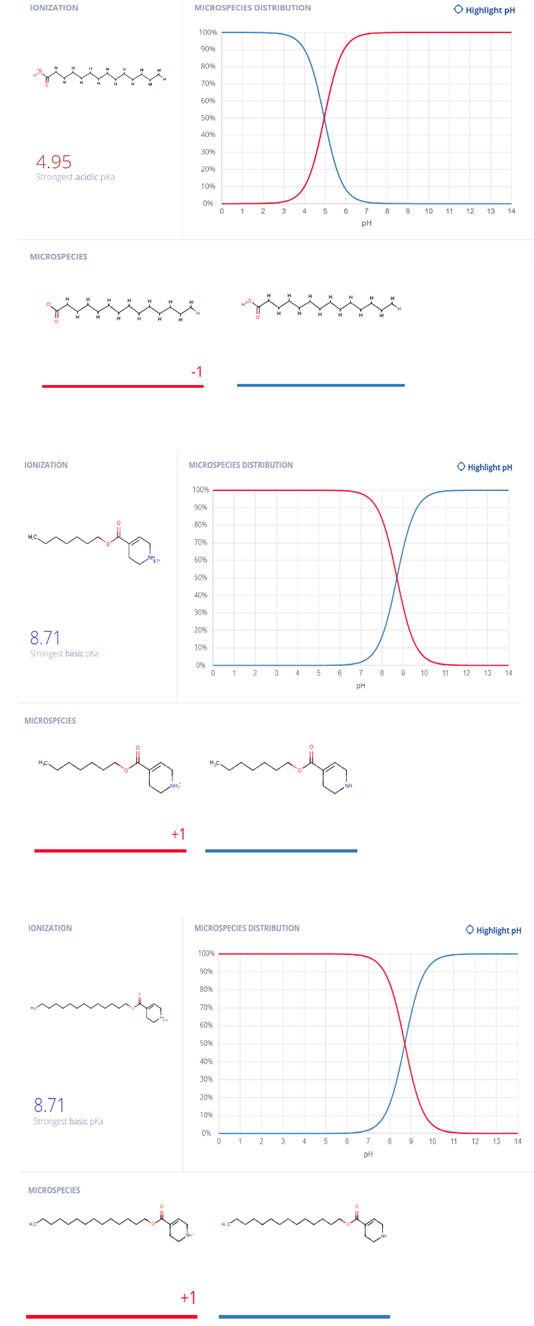


**Figure S5.** *In silico* prediction of pK_a_ values for MYR, **E7**, and **E14** using ChemAxon Chemicalize.


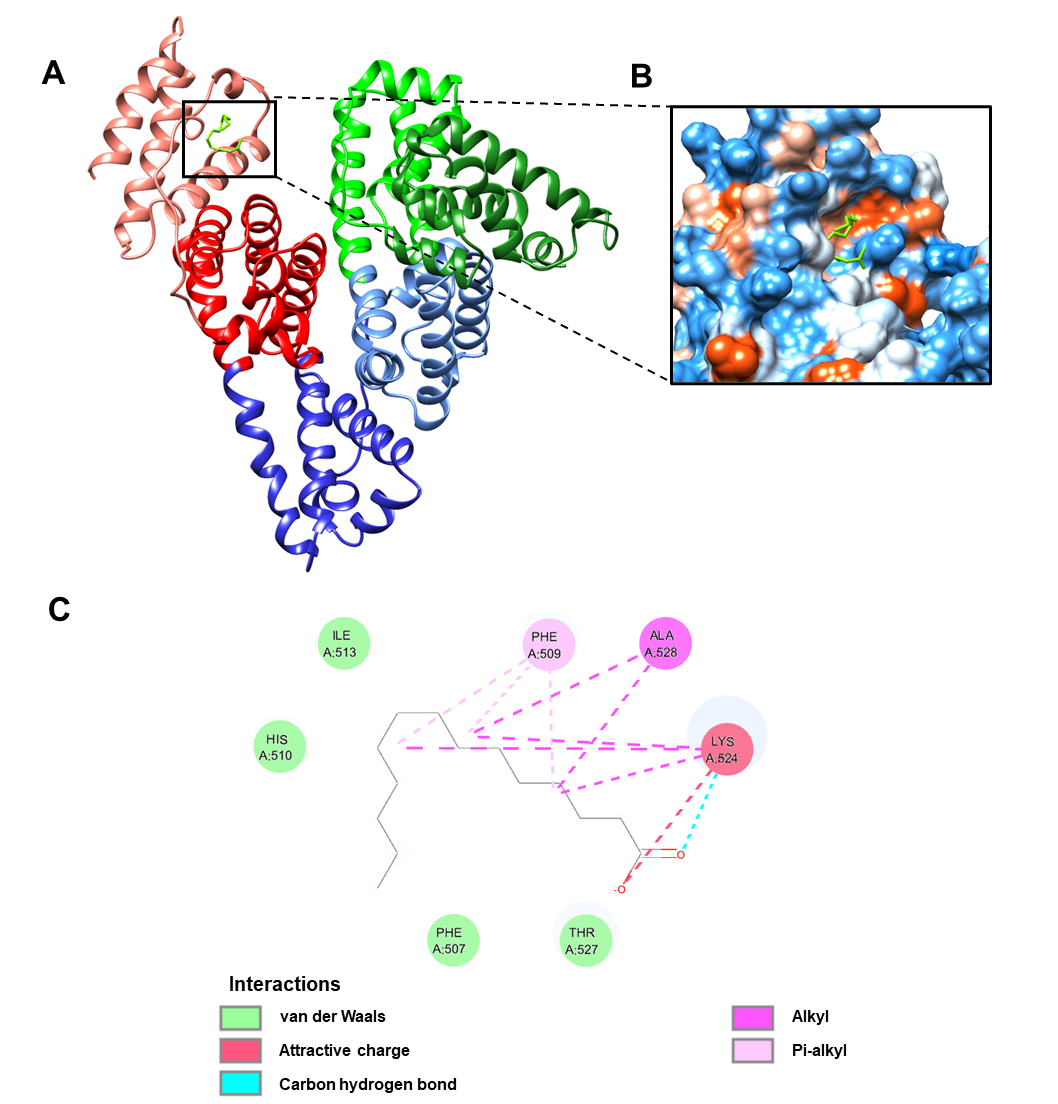


**Figure S6.** (A) Conformation with the lowest binding energy (–4.98 kcal/mol) of MYR (lime) docked onto the FA5 site of HSA. (B) Zoomed view highlighting the predicted binding pocket for MYR, shown through 3D surface model colored by hydrophobicity. (C) Schematic depiction of the various intermolecular interactions involved in the predicted HSA–MYR binding mode.


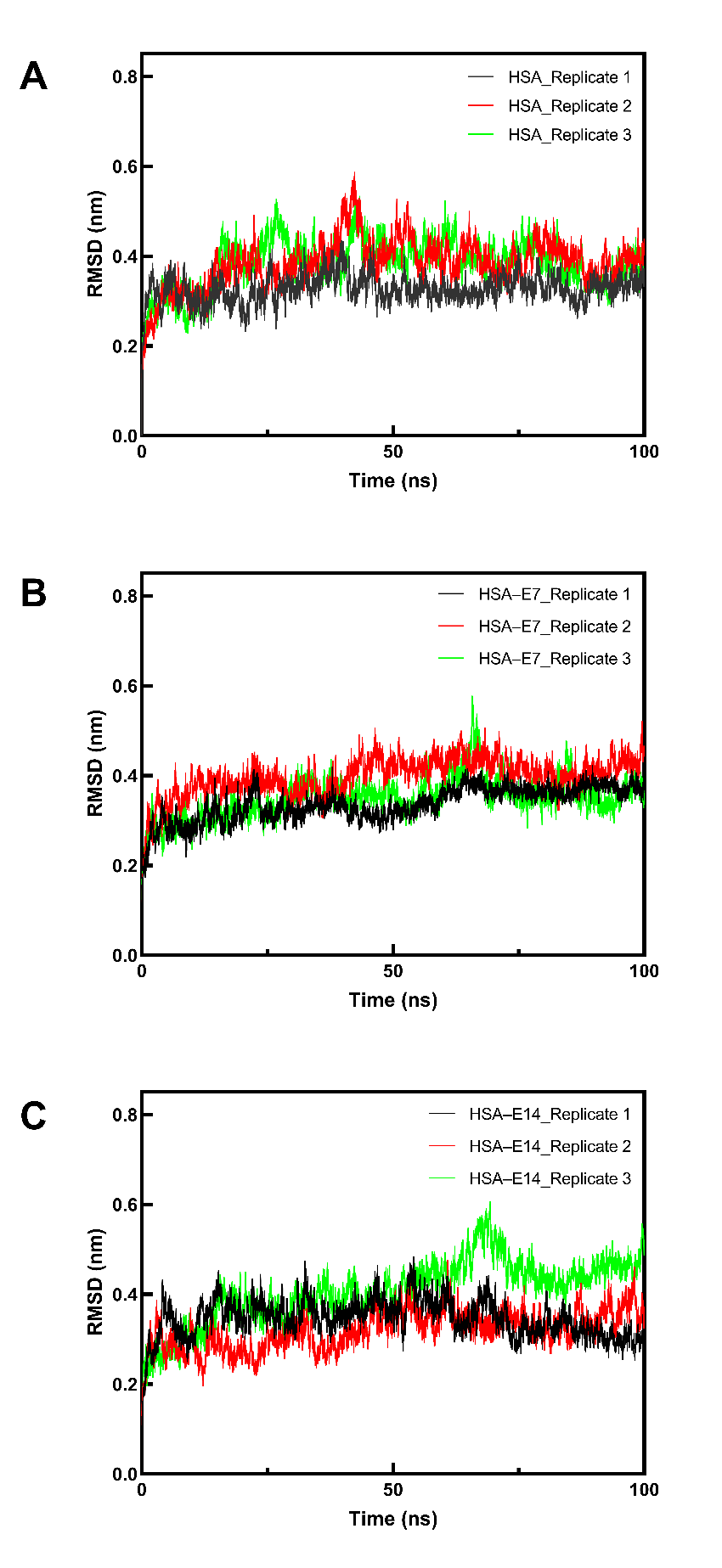


**Figure S7.** The RMSD of the HSA backbone after least-squares alignment in the (A) absence and in the presence of (B) **E7** and (C) **E14**. Each system was simulated in triplicate, with individual replicates shown as black, red, and green lines.


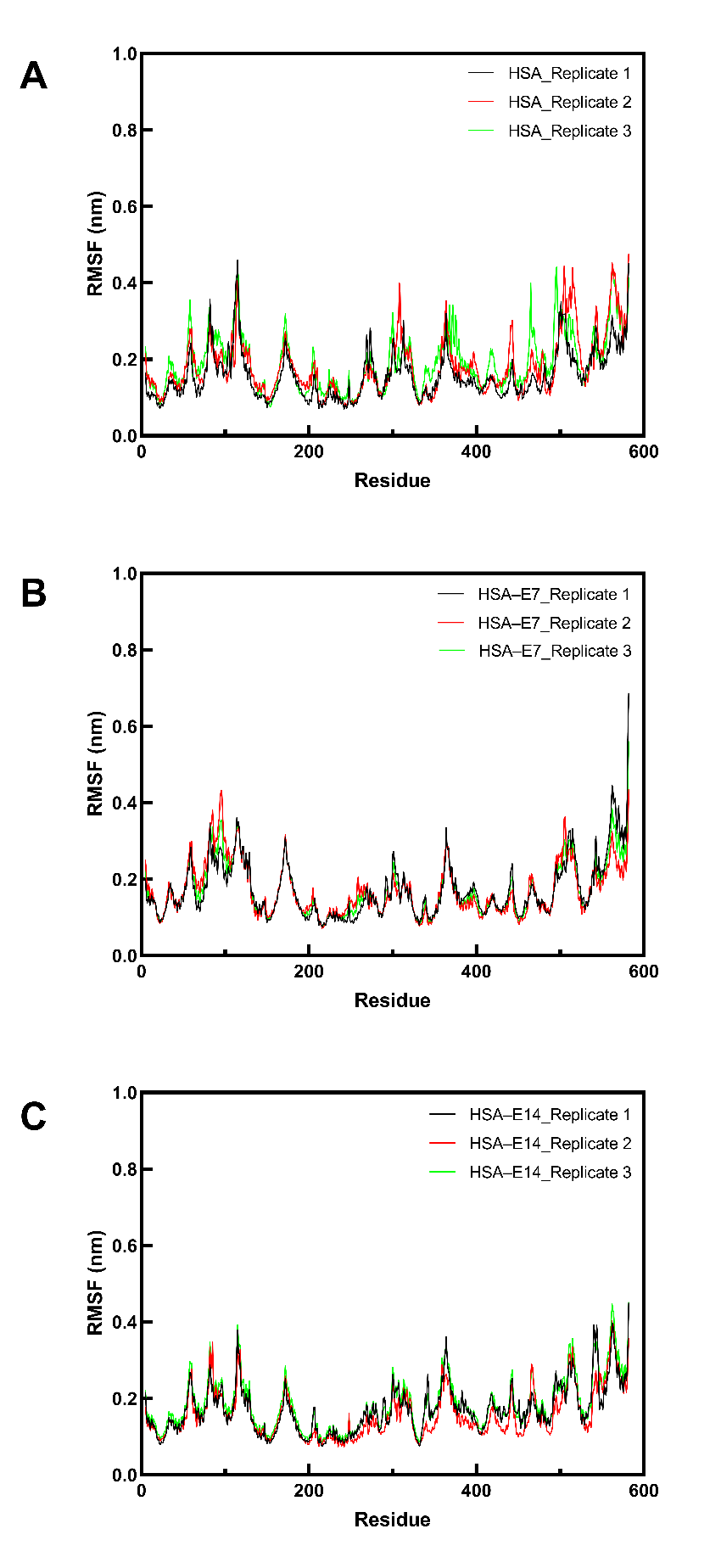


**Figure S8.** RMSF plots of HSA residues in the (A) absence and in the presence of (B) **E7** and (C) **E14**. Each system was simulated in triplicate, with individual replicates shown as black, red, and green lines.
